# Supplementary material for: Comparative proteomic analysis of the hemolymph and salivary glands of Rhodnius prolixus and R. colombiensis reveals candidates associated with differential lytic activity against Trypanosoma cruzi Dm28c and T. cruzi Y
Source: PLoS Negl Trop Dis. 2024 Apr 3;18(4):e0011452. doi: 10.1371/journal.pntd.0011452 (PMC10990223; doi:10.1371/journal.pntd.0011452)
Supplement: S1 Table — (DOCX) [file pntd.0011452.s001.docx]

**S1**. counts of parasite incubations with the saliva and hemolymph of *R. prolixus* and *R. colombiensis*

| Saliva*_R.prolixus* | | | | | | | | |
| --- | --- | --- | --- | --- | --- | --- | --- | --- |
|  | Hour 0 | | | | Hour 10 | | | |
| Epimastigotes_TcI | 30600000 | 24600000 | 26700000 | 24000000 | 29700000 | 19800000 | 24600000 | 28800000 |
| Negative_control_TcI | 26400000 | 24000000 | 33000000 | 26400000 | 29400000 | 18000000 | 30000000 | 29400000 |
| Epimastigotes_TcII | 20100000 | 27000000 | 28200000 | 27300000 | 8400000 | 14400000 | 18600000 | 15300000 |
| Negative_control_TcII | 27300000 | 30000000 | 32000000 | 27300000 | 29100000 | 26400000 | 34200000 | 29100000 |
| Positive control | 28000000 | 27600000 | 29800000 | 2580000 | 400000 | 150000 | 750000 | 150000 |
|  |  |  |  |  |  |  |  |  |
| Saliva_*R.colombiensis* | | | | | | | | |
|  | Hour 0 | | | | Hour 10 | | | |
| Epimastigotes_TcI | 29450000 | 35400000 | 36600000 | 29800000 | 29400000 | 33600000 | 34800000 | 30300000 |
| Negative_control_TcI | 28000000 | 31200000 | 35700000 | 29100000 | 31800000 | 28500000 | 36600000 | 30000000 |
| Epimastigotes_TcII | 28200000 | 28500000 | 30400000 | 28000000 | 24000000 | 30900000 | 29800000 | 23400000 |
| Negative_control_TcII | 28500000 | 27300000 | 30500000 | 25300000 | 30000000 | 29400000 | 31100000 | 25700000 |
| Positive control | 29200000 | 30100000 | 28000000 | 30000000 | 900000 | 600000 | 150000 | 400000 |

| Hemolymph_*Rprolixus* vs Epimastigotes | | | | | | |
| --- | --- | --- | --- | --- | --- | --- |
|  | Hour 0 | | | hour 14 | | |
| Epimastigotes_TcI | 25200000 | 26250000 | 26100000 | 24450000 | 25500000 | 27000000 |
| Negative control_TcI | 22200000 | 24600000 | 23850000 | 27750000 | 23550000 | 23700000 |
| Epimastigotes_TcII | 25200000 | 27150000 | 24000000 | 1650000 | 1200000 | 900000 |
| Negative control_TcII | 23550000 | 24300000 | 23100000 | 23700000 | 22650000 | 25800000 |
| Positive control | 25450000 | 26400000 | 26000000 | 600000 | 150000 | 0 |
|  |  |  |  |  |  |  |
|  | Hemolymph_*R_colombiensis* vs Epimastigotes | | | | | |
|  | Hour 0 | | | Hour 14 | | |
| Epimastigotes_TcI | 26000000 | 25500000 | 24900000 | 25500000 | 24500000 | 25900000 |
| Negative control_TcI | 25500000 | 25200000 | 26000000 | 25250000 | 24900000 | 25500000 |
| Epimastigotes_TcII | 25500000 | 24500000 | 24900000 | 25500000 | 25000000 | 25900000 |
| Negative control_TcII | 26500000 | 25200000 | 26000000 | 25500000 | 24900000 | 25500000 |
| Positive control | 26600000 | 25000000 | 26200000 | 750000 | 900000 | 150000 |

| Hemolymph_*R_prolixus* vs Trypomastigotes | | | | | | |
| --- | --- | --- | --- | --- | --- | --- |
|  | Hour 0 | | | Hour 14 | | |
| Trypomastigotes_TcI | 27300000 | 26400000 | 27300000 | 27000000 | 25500000 | 25650000 |
| Negative control_TcI | 26100000 | 25800000 | 27300000 | 25500000 | 26100000 | 26100000 |
| Trypomastigotes_TcII | 25200000 | 26400000 | 25800000 | 150000 | 450000 | 600000 |
| Negative control_TcII | 23700000 | 27000000 | 27600000 | 25800000 | 26250000 | 27000000 |
| Positive control | 27200000 | 29000000 | 28000000 | 750000 | 900000 | 600000 |
|  |  |  |  |  |  |  |
|  | Hemolymph_*R_colombiensis* vs Trypomastigotes | | | | | |
|  | Hour 0 | | | Hour 14 | | |
| Trypomastigotes_TcI | 29250000 | 27600000 | 25950000 | 25200000 | 24900000 | 32100000 |
| Negative control_TcI | 22200000 | 24600000 | 23850000 | 27750000 | 23550000 | 23700000 |
| Trypomastigotes_TcII | 22500000 | 23700000 | 28350000 | 21450000 | 24300000 | 26700000 |
| Negative control_TcII | 23550000 | 24300000 | 23100000 | 23700000 | 22650000 | 25800000 |
| Positive control | 30000000 | 28000000 | 27200000 | 600000 | 400000 | 150000 |
