# Supplementary material for: Comparative proteomic analysis of the hemolymph and salivary glands of Rhodnius prolixus and R. colombiensis reveals candidates associated with differential lytic activity against Trypanosoma cruzi Dm28c and T. cruzi Y
Source: PLoS Negl Trop Dis. 2024 Apr 3;18(4):e0011452. doi: 10.1371/journal.pntd.0011452 (PMC10990223; doi:10.1371/journal.pntd.0011452)
Supplement: S2 Table — (DOCX) [file pntd.0011452.s002.docx]

**S2 Table.** Common proteins detected of *R. prolixus* and *R. colombiensis* hemolymph, detected only in *R. prolixus* hemolymph, or detected only in *R. colombiensis.*

| **N°** | **Name** | **ID Uniprot** | ***Rpro*** | ***Rcol*** | **N°** | **Name** | **ID Uniprot** | ***Rpro*** | ***Rcol*** |
| --- | --- | --- | --- | --- | --- | --- | --- | --- | --- |
| 1 | Actin-5c | A0A023F5W2 | **√** | **√** | 69 | Retinoid-and fatty acid-binding glycoprotein variant PD | A0A170YQI4 | **√** | **√** |
| 2 | Aminopeptidase | A0A0V0G4Q6 | **√** | **√** | 70 | Serine-type endopeptidase activity | T1H815 | **√** | **√** |
| 3 | Angiotensin-converting enzyme | T1I107 | **√** | **√** | 71 | Sulfhydryl oxidase | T1I7C5 | **√** | **√** |
| 4 | Beta-galactosidase | R4G561 | **√** | **√** | 72 | Transferrin | B8LJ43 | **√** | **√** |
| 5 | Beta-GRP | B8LJ39 | **√** | **√** | 73 | Transferrin | A0A170Y4Q2 | **√** | **√** |
| 6 | Carboxylic ester hydrolase | A0A170XK23 | **√** | **√** | 74 | Tubulin alpha chain | A0A023F9Q4 | **√** | **√** |
| 7 | Carboxylic ester hydrolase | A0A0P4VP57 | **√** | **√** | 75 | Tubulin beta chain | A0A069DU54 | **√** | **√** |
| 8 | Carboxylic ester hydrolase | T1HW15 | **√** | **√** | 76 | Venom acid phosphatase acph-1-like protein isoform x2 | A0A170XK51 | **√** | **√** |
| 9 | Carboxypeptidase | R4FLC5 | **√** | **√** | 77 | Vitellogenin-6-like protein | A0A170XJA4 | **√** | **√** |
| 10 | Chemosensory protein | T1IAF9 | **√** | **√** | 78 | Putative catalytically inactive chitinase-like lectin | A0A0P4VGP5 | **√** | **√** |
| 11 | Cystatin | R4FP01 | **√** | **√** | 79 | Tryp_Spc | T1I2L3 | **√** | **√** |
| 12 | Ferritin | T1H8P8 | **√** | **√** | 80 | Tryp_Spc | T1HMX6 | **√** | **√** |
| 13 | Ferritin | T1HYY6 | **√** | **√** | 81 | Tryp_SPc/CLIP | T1I0A9 | **√** | **√** |
| 14 | Fructose-bisphosphate aldolase | T1I0J6 | **√** | **√** | 82 | Putative trypsin-like serine protease | A0A0P4VLU5 | **√** | **√** |
| 15 | Gelsolin-related actin binding protein | A0A161MIR2 | **√** | **√** | 83 | Serine-type endopeptidase activity | T1H816 | **√** | **√** |
| 16 | Heme-binding protein | Q8T5U0 | **√** | **√** | 84 | Serpin | T1IF83 | **√** | **√** |
| 17 | Lipophorin | T1HDK5 | **√** | **√** | 85 | vWFA super family | T1IFN4 | **√** | **√** |
| 18 | Phenol oxidase | T1HW22 | **√** | **√** | 86 | ML super family | T1HU92 | **√** | **√** |
| 19 | Phenol oxidase 1 | A0A1B2G385 | **√** | **√** | 87 | Prolixin antimicrobial peptide | B8QEI8 | **√** | **√** |
| 20 | Phenol oxidase 2 | A0A1B2G381 | **√** | **√** | 88 | Lysozyme | R4FPH4 | **√** | **√** |
| 21 | Phenol oxidase 2 | A0A1B2G381 | **√** | **√** | 89 | Putative gamma interferon inducible lysosomal thiol reductase gilt | A0A0P4VM92 | **√** | **√** |
| 22 | Protein with 4 pacifastin inhibitor domains lcmii | R4G3U6 | **√** | **√** | 90 | Putative gamma-interferon-inducible lysosomal thiol reductase | R4G4A3 | **√** | **√** |
| 23 | Putative actin muscle isoform x2 | A0A0P4VVE9 | **√** | **√** | 91 | I-set | T1HT36 | **√** | **√** |
| 24 | Putative aicar transformylase/imp cyclohydrolase/methylglyoxal synthase | A0A0P4VXM9 | **√** | **√** | 92 | IG_like | T1I8U8 | **√** | **√** |
| 25 | Putative aminopeptidase | A0A0P4VXT0 | **√** | **√** | 93 | Carboxylic ester hydrolase | T1HID5 | **√** |  |
| 26 | Putative apolipophorin-III | A0A170YUN3 | **√** | **√** | 94 | Carboxylic ester hydrolase | R4G810 | **√** |  |
| 27 | Putative apolipoprotein d | R4G3B5 | **√** | **√** | 95 | Carboxylic ester hydrolase | T1HGC0 | **√** |  |
| 28 | Putative arginine kinase | R4G5I9 | **√** | **√** | 96 | Ferritin | T1HTF0 | **√** |  |
| 29 | Putative beta-n-acetylglucosaminidase nag2 | R4G8V6 | **√** | **√** | 97 | Ferritin | R4G347 | **√** |  |
| 30 | Putative beta-n-acetylglucosaminidase nag2 | R4G8V6 | **√** | **√** | 98 | Ferritin | R4G4L4 | **√** |  |
| 31 | Putative c1q domain protein | R4FJF3 | **√** | **√** | 99 | Ferritin | R4FL73 | **√** |  |
| 32 | Putative carbonic anhydrase-like protein | A0A0P4VL85 | **√** | **√** | 100 | Larval serum protein 2 | A0A161MI44 | **√** |  |
| 33 | Putative cathepsin l | R4G4T0 | **√** | **√** | 101 | Lysozyme | A9LN32 | **√** |  |
| 34 | Putative chitinase | R4G8S4 | **√** | **√** | 102 | Nitrophorin 4A | Q7YT15 | **√** |  |
| 35 | Putative conserved secreted protein | A0A0P4VIB4 | **√** | **√** | 103 | Nitrophorin-1 | Q26239 | **√** |  |
| 36 | Putative esterase and lipase | A0A0P4VGT4 | **√** | **√** | 104 | Nitrophorin-4 | Q94734 | **√** |  |
| 37 | Putative fatty acyl-coa hydrolase medium chain | A0A0P4VL69 | **√** | **√** | 105 | Nucleoside diphosphate kinase | A0A0P4VSJ7 | **√** |  |
| 38 | Putative fibrillin | R4FJG3 | **√** | **√** | 106 | Odorant-binding protein | Q7YSZ4 | **√** |  |
| 39 | Putative galactoside-binding lectin | A0A0P4VKZ1 | **√** | **√** | 107 | Putative aldehyde dehydrogenase | A0A0P4VVF1 | **√** |  |
| 40 | Putative gamma-glutamyl hydrolase | A0A0P4VNM6 | **√** | **√** | 108 | Putative calsyntenin | A0A023FAK9 | **√** |  |
| 41 | Putative gamma-glutamyl hydrolase | A0A0P4VTW3 | **√** | **√** | 109 | Putative cu/zn-superoxide dismutase | A0A0P4VG48 | **√** |  |
| 42 | Putative glutathione s-transferase | A0A0P4VFN3 | **√** | **√** | 110 | Putative cysteine proteinase cathepsin l | R4G406 | **√** |  |
| 43 | Putative hexamerin | A0A0P4W1R7 | **√** | **√** | 111 | Putative defensin | A0A0P4VLV7 | **√** |  |
| 44 | Putative hexamerin | A0A0V0GDS7 | **√** | **√** | 112 | Putative glycine-rich cuticle protein | A0A0P4VJT1 | **√** |  |
| 45 | Putative hsp70 protein | R4FQG8 | **√** | **√** | 113 | Putative juvenile hormone | A0A0P4VQZ5 | **√** |  |
| 46 | Putative insect pheromone-binding family | A0A0P4VJD4 | **√** | **√** | 114 | Putative m28 zn-peptidase glutaminyl cyclase | A0A0P4VJW6 | **√** |  |
| 47 | Putative i-type lysozyme 3 | A0A0P4VPP9 | **√** | **√** | 115 | Putative odorant binding protein | R4G2S7 | **√** |  |
| 48 | Putative low density lipoprotein receptor | A0A0P4VLG8 | **√** | **√** | 116 | Putative secreted protein | A0A0P4VJV5 | **√** |  |
| 49 | Putative lysosomal & prostatic acid phosphatase | A0A0P4VH19 | **√** | **√** | 117 | Putative secreted protein | A0A0P4VL09 | **√** |  |
| 50 | Putative major royal jelly protein | A0A0P4VLE7 | **√** | **√** | 118 | Putative teneurin-1 | A0A023F492 | **√** |  |
| 51 | Putative neural cell adhesion molecule 2-like protein | A0A0P4VPG5 | **√** | **√** | 119 | Putative trypsin-like serine protease | R4G5A7 | **√** |  |
| 52 | Putative odorant-binding protein | R4G3B3 | **√** | **√** | 120 | SVWC | T1HG60 | **√** |  |
| 53 | Putative odorant-binding protein 1 obp obp | A0A0P4VRJ7 | **√** | **√** | 121 | Tubulin beta chain | R4G4U5 |  | **√** |
| 54 | Putative paramyosin | A0A0P4VYX5 | **√** | **√** | 122 | Transgelin | A0A0P4VTZ7 |  | **√** |
| 55 | Putative phosphoribosylaminoimidazole carboxylase | R4FNG8 | **√** | **√** | 123 | Isocitrate dehydrogenase [NADP] | A0A0P4VZE6 |  | **√** |
| 56 | Putative prolylcarboxypeptidase | R4G5X2 | **√** | **√** | 124 | Retinoid-and fatty acid-binding glycoprotein variant C | A0A170YQL2 |  | **√** |
| 57 | Putative prophenoloxidase | A0A0V0G7Q5 | **√** | **√** | 125 | Alpha-1,2-Mannosidase | A0A0P4W0H0 |  | **√** |
| 58 | Putative salivary lipocalin | R4FN82 | **√** | **√** | 126 | Odorant-binding protein RproOBP6 | C5J8H2 |  | **√** |
| 59 | Putative salivary lipocalin | A0A0P4VR25 | **√** | **√** | 127 | Putative multifunctional chaperone | A0A069DQN5 |  | **√** |
| 60 | Putative salivary serpin | A0A069DZE0 | **√** | **√** | 128 | Putative actin regulatory gelsolin/villin family | A0A0P4VVN5 |  | **√** |
| 61 | Putative serine carboxypeptidase | R4G841 | **√** | **√** | 129 | Putative enolase length | R4G4U2 |  | **√** |
| 62 | Putative serine proteinase inhibitor | A0A023EZF2 | **√** | **√** | 130 | Putative actin-depolymerizing factor 1 | A0A023F9W1 |  | **√** |
| 63 | Putative serpin length | R4FLP4 | **√** | **√** | 131 | Putative laminin g domain protein | A0A0P4VNA5 |  | **√** |
| 64 | Putative triabin-like lipocalin | R4FLZ0 | **√** | **√** | 132 | Putative inter-alpha-trypsin inhibitor heavy chain h4-like protein | A0A069DVF3 |  | **√** |
| 65 | Putative triabin-like lipocalin 4a | R4G4J2 | **√** | **√** | 133 | Putative prokaryotic long-chain fatty acid coa synthetase | A0A0V0G4H2 |  | **√** |
| 66 | Putative vitellogenin | R4G3Y1 | **√** | **√** | 134 | Putative beta-1 | A0A023F500 |  | **√** |
| 67 | Putative vitellogenin | A0A0P4VS21 | **√** | **√** | 135 | Putative carboxypeptidase a | R4G8G2 |  | **√** |
| 68 | Putative vitellogenin-6-like protein | A0A0P4VJ72 | **√** | **√** |  |  |  |  |  |
