# Supplementary material for: Comparative proteomic analysis of the hemolymph and salivary glands of Rhodnius prolixus and R. colombiensis reveals candidates associated with differential lytic activity against Trypanosoma cruzi Dm28c and T. cruzi Y
Source: PLoS Negl Trop Dis. 2024 Apr 3;18(4):e0011452. doi: 10.1371/journal.pntd.0011452 (PMC10990223; doi:10.1371/journal.pntd.0011452)
Supplement: S3 Table — (DOCX) [file pntd.0011452.s003.docx]

**S3 Table.** Common proteins detected in the salivary glands of *R. prolixus* and *R. colombiensis*, detected only in *R. prolixus*, or detected only in *R. colombiensis.*

| N° | **Name** | **ID Uniprot** | ***Rpro*** | ***Rcol*** | N° | **Name** | **ID Uniprot** | ***Rpro*** | ***Rcol*** |
| --- | --- | --- | --- | --- | --- | --- | --- | --- | --- |
| 1 | Transferrin | B8LJ43 | **√** | **√** | 605 | Uncharacterized protein | T1HYV6 | **√** | **√** |
| 2 | Uncharacterized protein | T1HU08 | **√** | **√** | 606 | Putative nucleic acid-binding protein | A0A0P4VNT1 | **√** | **√** |
| 3 | Nitrophorin 4A | Q7YT15 | **√** | **√** | 607 | Putative calmodulin | A0A023F9W6 | **√** | **√** |
| 4 | Lipophorin | T1HDK5 | **√** | **√** | 608 | Uncharacterized protein | T1IAP8 | **√** | **√** |
| 5 | Nitrophorin-2 | Q26241 | **√** | **√** | 609 | Putative pyridoxal 5-phosphate | A0A0P4VIK4 | **√** | **√** |
| 6 | Nitrophorin-4 | Q94734 | **√** | **√** | 610 | Putative adaptor protein enigma | A0A0P4W4W1 | **√** | **√** |
| 7 | Putative salivary platelet aggregation inhibitor 1 | R4FPG4 | **√** | **√** | 611 | Putative small gtpase of the ras superfamily | A0A023F5P8 | **√** | **√** |
| 8 | Putative chitinase | R4G8S4 | **√** | **√** | 612 | Peptidyl-prolyl cis-trans isomerase | T1HVU3 | **√** | **√** |
| 9 | Putative nitrophorin 1a | R4FPW7 | **√** | **√** | 613 | Sideroflexin | A0A0P4VLD5 | **√** | **√** |
| 10 | Nitrophorin-3 | Q94733 | **√** | **√** | 614 | Putative histone h1 | R4G5F7 | **√** | **√** |
| 11 | Putative actin muscle isoform x2 | A0A0P4VVE9 | **√** | **√** | 615 | Putative ubiquinone oxidoreductase ndufb9/b22 subunit | A0A0P4VSP6 | **√** | **√** |
| 12 | Lipocalin AI-4 | Q7YT09 | **√** | **√** | 616 | Putative nadh-ubiquinone oxidoreductase nufs7/psst/20 kDa subunit | A0A0P4VQ19 | **√** | **√** |
| 13 | Biogenic amine-binding protein | Q86PT9 | **√** | **√** | 617 | 60S ribosomal protein L29 | A0A069DNP0 | **√** | **√** |
| 14 | Protein disulfide-isomerase | A0A0P4VT63 | **√** | **√** | 618 | Uncharacterized protein | T1I1H6 | **√** | **√** |
| 15 | Putative scp / tpx-1 / ag5 / pr-1 / sc7 family of extracellular | A0A0P4VNH0 | **√** | **√** | 619 | Putative peptide chain release factor 1 erf1 | A0A069DZ03 | **√** | **√** |
| 16 | Putative hsp70 protein | R4FQG8 | **√** | **√** | 620 | Putative nadh-ubiquinone oxidoreductase subunit ndufb10/pdsw | A0A0P4VN49 | **√** | **√** |
| 17 | Tubulin beta chain | R4G4U5 | **√** | **√** | 621 | alpha-1,2-Mannosidase | A0A0P4VUB2 | **√** | **√** |
| 18 | Uncharacterized protein | T1IF83 | **√** | **√** | 622 | Putative small nuclear ribonucleoprotein snrnp sm core protein | A0A0P4VLF7 | **√** | **√** |
| 19 | Elongation factor 1-alpha | A0A069DZ22 | **√** | **√** | 623 | Putative ran gtpase-activating protein 1 | A0A0P4VTS9 | **√** | **√** |
| 20 | Nitrophorin-1 | Q26239 | **√** | **√** | 624 | Uncharacterized protein | A0A069DV10 | **√** | **√** |
| 21 | ATP synthase subunit beta | R4FQF4 | **√** | **√** | 625 | Anamorsin homolog | R4G435 | **√** | **√** |
| 22 | Uncharacterized protein | T1I7K2 | **√** | **√** | 626 | NADH dehydrogenase [ubiquinone] 1 subunit C2 | A0A0P4VMY5 | **√** | **√** |
| 23 | Putative triabin length | R4G339 | **√** | **√** | 627 | Tubulin beta chain | A0A069DU54 | **√** | **√** |
| 24 | Putative arginine kinase | R4G5I9 | **√** | **√** | 628 | Protein disulfide-isomerase | A0A0P4VRU8 | **√** | **√** |
| 25 | Uncharacterized protein | T1HJT8 | **√** | **√** | 629 | Annexin | R4G8E5 | **√** | **√** |
| 26 | ATP synthase subunit alpha | R4FQG1 | **√** | **√** | 630 | Putative heat shock 70 kDa protein cognate | A0A023F5I3 | **√** | **√** |
| 27 | Tubulin alpha chain | A0A023F9Q4 | **√** | **√** | 631 | Aconitate hydratase, mitochondrial | A0A0P4VIL1 | **√** | **√** |
| 28 | Uncharacterized protein | T1IAB6 | **√** | **√** | 632 | Putative coproporphyrinogen iii oxidase cpo/hem13 | A0A0P4VQY7 | **√** | **√** |
| 29 | Putative elongation factor 2 | R4FQU1 | **√** | **√** | 633 | Putative 40s ribosomal protein s3 | A0A0P4VP84 | **√** | **√** |
| 30 | Catalase | A0A0P4VT66 | **√** | **√** | 634 | p450 enzyme | Q7YSZ3 | **√** | **√** |
| 31 | Putative hsp90 protein | R4FMH8 | **√** | **√** | 635 | Putative beta-n-acetylglucosaminidase nag2 | R4G8V6 | **√** | **√** |
| 32 | Putative aminopeptidase | A0A0P4VXT0 | **√** | **√** | 636 | Putative vacuolar h+-atpase v1 sector subunit b | A0A0N7Z9E6 | **√** | **√** |
| 33 | Putative transitional endoplasmic reticulum atpase ter94 | A0A0P4VTA9 | **√** | **√** | 637 | Annexin | A0A0P4VWP5 | **√** | **√** |
| 34 | Fructose-bisphosphate aldolase | T1I0J6 | **√** | **√** | 638 | Uncharacterized protein Triatoma rubida | G8JKF2 | **√** | **√** |
| 35 | Uncharacterized protein | T1I1L9 | **√** | **√** | 639 | Putative prohibitin-like protein | A0A0N7Z9D5 | **√** | **√** |
| 36 | Putative endoplasmic reticulum glucose-regulated protein grp94/endoplasmin | R4FQH4 | **√** | **√** | 640 | 40S ribosomal protein S3a | A0A0P4VV23 | **√** | **√** |
| 37 | Dolichyl-diphosphooligosaccharide--protein glycosyltransferase subunit 1 | R4FP91 | **√** | **√** | 641 | Ferritin | R4G8K8 | **√** | **√** |
| 38 | Putative: similar to profilin | R4FQ51 | **√** | **√** | 642 | 40S ribosomal protein S8 | A0A0N7Z9C6 | **√** | **√** |
| 39 | Annexin | T1IC12 | **√** | **√** | 643 | Uncharacterized protein | T1HVV8 | **√** | **√** |
| 40 | Aconitate hydratase, mitochondrial | R4G408 | **√** | **√** | 644 | Putative multifunctional chaperone 14-3-3 family | A0A0P4VWN9 | **√** | **√** |
| 41 | Putative heat shock 70 kDa protein cognate 5 | A0A0P4VNA9 | **√** | **√** | 645 | Putative peroxiredoxin posttranslational modification | A0A0P4VHJ7 | **√** | **√** |
| 42 | Delta-aminolevulinic acid dehydratase | R4G3N4 | **√** | **√** | 646 | ATP synthase subunit gamma | T1HN70 | **√** | **√** |
| 43 | Putative glutathione s-transferase | A0A0P4VFN3 | **√** | **√** | 647 | Putative tata-binding protein-interacting protein | A0A023F571 | **√** | **√** |
| 44 | Putative enoyl-coa hydratase | A0A0P4W0W1 | **√** | **√** | 648 | Putative polyc-binding hnrnp-k protein hrb57a/hnrnp | A0A069DS29 | **√** | **√** |
| 45 | Putative transcriptional coactivator | R4FNW9 | **√** | **√** | 649 | Putative ribosomal protein s2 | A0A023F9B5 | **√** | **√** |
| 46 | Carboxypeptidase | R4FLC5 | **√** | **√** | 650 | 40s ribosomal protein s16 | A0A171B9P1 | **√** | **√** |
| 47 | Cytochrome P450 (CYP4D) | T1HL45 | **√** | **√** | 651 | Putative 40s ribosomal protein s7 | A0A069DQ79 | **√** | **√** |
| 48 | Putative molecular chaperone grp170/sil1 hsp70 superfamily protein | A0A0P4VS14 | **√** | **√** | 652 | Coatomer subunit delta | A0A0P4VZW3 | **√** | **√** |
| 49 | Putative paramyosin | A0A0P4VYX5 | **√** | **√** | 653 | Putative chaperonin subunit 6a zeta | A0A0P4VZV8 | **√** | **√** |
| 50 | Aconitate hydratase | A0A0P4VTU7 | **√** | **√** | 654 | Putative l-3-hydroxyacyl-coenzyme a dehydrogenase | R4G8E3 | **√** | **√** |
| 51 | Putative salivary protein mys2 | A0A0P4VQE4 | **√** | **√** | 655 | Putative cytochrome c | A0A069DVF4 | **√** | **√** |
| 52 | Putative glutamate dehydrogenase | R4G3J2 | **√** | **√** | 656 | Putative cytoskeletal protein adducin | A0A0P4VWP8 | **√** | **√** |
| 53 | Actin-5c | A0A023F5W2 | **√** | **√** | 657 | Putative glutamate synthase | A0A0P4VLV2 | **√** | **√** |
| 54 | Uncharacterized protein | T1I795 | **√** | **√** | 658 | Putative 26s protease regulatory subunit 6b | A0A0P4VYP8 | **√** | **√** |
| 55 | Putative hydroxyacyl-coa dehydrogenase/enoyl-coa hydratase | A0A0P4VTI8 | **√** | **√** | 659 | Putative isoleucyl-trna synthetase | A0A0P4VKI1 | **√** | **√** |
| 56 | Sodium/potassium-transporting ATPase subunit alpha | A0A023F5Y6 | **√** | **√** | 660 | Uncharacterized protein | T1IA61 | **√** | **√** |
| 57 | Putative ubiquitin/60s ribosomal protein l40 fusion | A0A023F9R9 | **√** | **√** | 661 | Putative two dm9 repeat protein | R4FL58 | **√** | **√** |
| 58 | Putative transketolase | A0A0P4VWT3 | **√** | **√** | 662 | Putative 60s ribosomal protein l8 | A0A069DY45 | **√** | **√** |
| 59 | Putative multifunctional chaperone | A0A069DQN5 | **√** | **√** | 663 | Kinesin-like protein | T1I4R3 | **√** | **√** |
| 60 | Putative moesin/ezrin/radixin protein 1 | A0A0P4VUQ0 | **√** | **√** | 664 | Putative 26s proteasome regulatory complex | R4FP59 | **√** | **√** |
| 61 | Putative short-chain alcohol dehydrogenase/3-hydroxyacyl-coa dehydrogenase | A0A0P4VTY5 | **√** | **√** | 665 | Proteasome subunit alpha type | A0A069DQZ8 | **√** | **√** |
| 62 | Putative calreticulin | R4G8F8 | **√** | **√** | 666 | Uncharacterized protein | T1HJS8 | **√** | **√** |
| 63 | Uncharacterized protein | T1HRG2 | **√** | **√** | 667 | Putative 60s ribosomal protein l14 | R4G883 | **√** | **√** |
| 64 | Protein disulfide-isomerase | T1HYN9 | **√** | **√** | 668 | Nucleoplasmin-like protein isoform | A0A023F9L2 | **√** | **√** |
| 65 | Putative ribosomal protein l4 cg5502-pa isoform 1 | R4FMG4 | **√** | **√** | 669 | Proteasome subunit alpha type | A0A0P4VNA4 | **√** | **√** |
| 66 | Putative prohibitin | A0A0P4VUP1 | **√** | **√** | 670 | Putative translational activator gcn1 | A0A023FAI4 | **√** | **√** |
| 67 | Ferritin | A0A0P4VY94 | **√** | **√** | 671 | Putative ras-related protein rab-7a-like isoform 1 | A0A023FAJ8 | **√** | **√** |
| 68 | Putative vigilin | A0A023FAG6 | **√** | **√** | 672 | Putative 26s proteasome regulatory complex atpase rpt2 | A0A023FCX8 | **√** | **√** |
| 69 | Ferritin | T1H8P8 | **√** | **√** | 673 | 60S ribosomal protein L18a | A0A069DQ01 | **√** | **√** |
| 70 | Polyadenylate-binding protein | A0A023FCX3 | **√** | **√** | 674 | Eukaryotic translation initiation factor 3 subunit C | A0A0P4W0S5 | **√** | **√** |
| 71 | Citrate synthase | A0A0P4VSB3 | **√** | **√** | 675 | NADH dehydrogenase [ubiquinone] flavoprotein 1, mitochondrial | A0A0P4VQI4 | **√** | **√** |
| 72 | Uncharacterized protein | T1HRT1 | **√** | **√** | 676 | 40S ribosomal protein S12 | A0A069DPB2 | **√** | **√** |
| 73 | Uroporphyrinogen decarboxylase | A0A0P4VNE6 | **√** | **√** | 677 | Putative rnase l inhibitor abc superfamily protein | A0A0N7Z9B3 | **√** | **√** |
| 74 | Putative actin regulatory gelsolin/villin family | A0A0P4VVN5 | **√** | **√** | 678 | Putative programmed cell death protein | A0A0P4VUE5 | **√** | **√** |
| 75 | Uncharacterized protein | T1I122 | **√** | **√** | 679 | Uncharacterized protein | T1H8D0 | **√** | **√** |
| 76 | Uncharacterized protein | T1H969 | **√** | **√** | 680 | Uncharacterized protein | T1IDG3 | **√** | **√** |
| 77 | Malate dehydrogenase | R4G866 | **√** | **√** | 681 | Putative glutathione s-transferase | A0A0P4VUE9 | **√** | **√** |
| 78 | 40S ribosomal protein S3a | A0A161MTG7 | **√** | **√** | 682 | Putative leucine-rich acidic nuclear protein | A0A0P4VPG8 | **√** | **√** |
| 79 | Putative lipocalin | R4G376 | **√** | **√** | 683 | Putative 60s ribosomal protein l26 | A0A069DW40 | **√** | **√** |
| 80 | Putative ras-related protein rab-1a | A0A0P4VKJ7 | **√** | **√** | 684 | Putative histidyl-trna synthetase | A0A0P4VPA7 | **√** | **√** |
| 81 | Ferrochelatase | A0A0P4VTD1 | **√** | **√** | 685 | MICOS complex subunit MIC60 | A0A0P4VUY0 | **√** | **√** |
| 82 | Succinyl-CoA:3-ketoacid-coenzyme A transferase | A0A0P4VV86 | **√** | **√** | 686 | Putative carbonic anhydrase 2-like protein | A0A0P4VHZ4 | **√** | **√** |
| 83 | Putative beta-spectrin | A0A0P4VTQ5 | **√** | **√** | 687 | Eukaryotic translation initiation factor 3 subunit I | R4FQ83 | **√** | **√** |
| 84 | Nucleoside diphosphate kinase | A0A0P4VSJ7 | **√** | **√** | 688 | Putative nucleotide excision repair factor nef2 rad23 component | A0A0P4VUW1 | **√** | **√** |
| 85 | Putative g protein | A0A0P4VYB2 | **√** | **√** | 689 | Putative 26s proteasome regulatory complex subunit rpn8/psmd7 | A0A069DSK6 | **√** | **√** |
| 86 | Putative vacuolar h+-atpase v1 sector subunit a | A0A0N7Z969 | **√** | **√** | 690 | Uncharacterized protein | A0A0P4VPQ3 | **√** | **√** |
| 87 | Putative aspartyl protease | R4G4V2 | **√** | **√** | 691 | Putative transcriptional regulator of the pur family single-stranded-dna-binding protein | A0A023F5M9 | **√** | **√** |
| 88 | Putative mitochondrial adp/atp carrier protein | R4FQ65 | **√** | **√** | 692 | Ribosomal protein L15 | A0A0P4VKP7 | **√** | **√** |
| 89 | Putative alkyl hydroperoxide reductase/peroxiredoxin | R4G2Z2 | **√** | **√** | 693 | S-(hydroxymethyl)glutathione dehydrogenase | A0A0P4VSC3 | **√** | **√** |
| 90 | Glucose-6-phosphate isomerase | A0A0P4VK76 | **√** | **√** | 694 | Putative 26s proteasome regulatory complex subunit rpn7/psmd6 | A0A0P4VUL5 | **√** | **√** |
| 91 | Uncharacterized protein | T1HZB4 | **√** | **√** | 695 | Putative rna-binding protein musashi/mrna cleavage and polyadenylation factor i complex | A0A0P4VMD1 | **√** | **√** |
| 92 | Putative 40s ribosomal protein s3 | A0A023FAI9 | **√** | **√** | 696 | Putative bifunctional atp sulfurylase/adenosine 5'-phosphosulfate kinase | A0A0P4VVW0 | **√** | **√** |
| 93 | Putative acetyl-coa hydrolase | A0A0P4VXC0 | **√** | **√** | 697 | Putative ribosomal protein | G1K0B7 | **√** | **√** |
| 94 | Putative elongation factor 1-gamma | R4FNR5 | **√** | **√** | 698 | Eukaryotic translation initiation factor 3 subunit M | R4G4J3 | **√** | **√** |
| 95 | Fibroblast growth factor receptor homolog 1 | R4FLP0 | **√** | **√** | 699 | 60S ribosomal protein L27 | A0A023F8Q2 | **√** | **√** |
| 96 | Putative serine protease 10 | A0A0N7Z8E7 | **√** | **√** | 700 | Proteasome subunit beta | A0A0P4VPG6 | **√** | **√** |
| 97 | T-complex protein 1 subunit delta | A0A0P4VQT8 | **√** | **√** | 701 | Putative mitochondrial carrier protein | R4FP18 | **√** | **√** |
| 98 | Clathrin heavy chain | A0A069DYF4 | **√** | **√** | 702 | Putative pyrazinamidase/nicotinamidase pnc1 | A0A023F4C6 | **√** | **√** |
| 99 | T-complex protein 1 subunit gamma | A0A0P4VYQ8 | **√** | **√** | 703 | Putative 26s proteasome regulatory complex subunit | A0A069DU92 | **√** | **√** |
| 100 | Uncharacterized protein | T1I3Q3 | **√** | **√** | 704 | Putative 40s ribosomal protein s11 | A0A069DPD9 | **√** | **√** |
| 101 | Uncharacterized protein | T1HS20 | **√** | **√** | 705 | Putative 26s proteasome regulatory complex atpase rpt5 | A0A069DUE0 | **√** | **√** |
| 102 | Uncharacterized protein | T1HRF6 | **√** | **√** | 706 | Putative mannose-1-phosphate guanyltransferase | R4G8S3 | **√** | **√** |
| 103 | Putative 3-hydroxybutyrate dehydrogenase type 2 | R4FQ30 | **√** | **√** | 707 | Uncharacterized protein | T1I6I8 | **√** | **√** |
| 104 | Annexin | R4G4I1 | **√** | **√** | 708 | Putative dna replication helicase | A0A023F5K8 | **√** | **√** |
| 105 | Uncharacterized protein | T1H8B1 | **√** | **√** | 709 | Putative eukaryotic translation initiation factor 2 subunit 3 y-linked-like protein | A0A069DZ29 | **√** | **√** |
| 106 | Putative 60s ribosomal protein l5 | A0A0P4VPV1 | **√** | **√** | 710 | Putative receptor mediating netrin-dependent axon guidance | A0A0P4VUL8 | **√** | **√** |
| 107 | Putative enolase length | R4G4U2 | **√** | **√** | 711 | Uncharacterized protein | A0A0N7Z9K7 | **√** | **√** |
| 108 | Putative 60s ribosomal protein l9-like isoform x2 | A0A0P4VRT6 | **√** | **√** | 712 | Putative cell division cycle and apoptosis regulator protein 1 | A0A0P4VMZ8 | **√** | **√** |
| 109 | Putative acyl-coa dehydrogenase | A0A0P4VIT3 | **√** | **√** | 713 | Succinate--CoA ligase [ADP-forming] subunit beta, mitochondrial | A0A0P4VNG2 | **√** | **√** |
| 110 | Putative electron transfer flavoprotein alpha subunit | A0A0P4VK70 | **√** | **√** | 714 | Putative ubiquinone oxidoreductase ndufs2/49 kDa subunit | A0A0P4VTV0 | **√** | **√** |
| 111 | Putative sugar kinase | A0A0N7Z998 | **√** | **√** | 715 | Putative adenine phosphoribosyltransferase | A0A0N7Z999 | **√** | **√** |
| 112 | Elongation factor Tu | A0A0P4VIJ7 | **√** | **√** | 716 | Putative arginyl-trna synthetase translation | A0A0P4VT38 | **√** | **√** |
| 113 | Putative 3-hydroxyisobutyrate dehydrogenase | R4FKE4 | **√** | **√** | 717 | Putative 60s ribosomal protein l32 | A0A069DN22 | **√** | **√** |
| 114 | Putative oligosaccharyltransferase | R4FP51 | **√** | **√** | 718 | Putative rna-binding protein lark | A0A069DYR2 | **√** | **√** |
| 115 | Putative chaperonin | A0A0P4VTR7 | **√** | **√** | 719 | Uncharacterized protein | T1HP88 | **√** | **√** |
| 116 | Putative metallopeptidase | R4G8R7 | **√** | **√** | 720 | Adenylyl cyclase-associated protein | A0A0P4VL56 | **√** | **√** |
| 117 | Putative 60s ribosomal protein l7a | A0A0P4VVP8 | **√** | **√** | 721 | Uncharacterized protein | T1IFE6 | **√** | **√** |
| 118 | FK506-binding protein | R4G5K8 | **√** | **√** | 722 | Putative g-protein coupled receptor | A0A0P4VPW0 | **√** | **√** |
| 119 | Peptidyl-prolyl cis-trans isomerase | A0A0P4VLQ3 | **√** | **√** | 723 | Putative adenylosuccinate synthase | A0A0P4VIL0 | **√** | **√** |
| 120 | Putative amidase | A0A0P4VTM9 | **√** | **√** | 724 | Uncharacterized protein | T1HBQ4 | **√** | **√** |
| 121 | Putative electron transfer flavoprotein beta subunit | A0A0P4VLG6 | **√** | **√** | 725 | Putative ribosome bioproteinsis protein | A0A0N7Z900 | **√** | **√** |
| 122 | Putative tumor protein d52 family | A0A0P4VHA6 | **√** | **√** | 726 | Putative translation initiation factor 4f helicase subunit eif-4a | A0A023F5Q2 | **√** | **√** |
| 123 | Putative chaperonin | A0A0P4VUP7 | **√** | **√** | 727 | Eukaryotic translation initiation factor 3 subunit F | A0A171A976 | **√** | **√** |
| 124 | Putative ribosomal protein s19e | R4FLX6 | **√** | **√** | 728 | Putative vesicle coat complex copii subunit sec13 | A0A0P4VLS4 | **√** | **√** |
| 125 | 40S ribosomal protein SA | A0A0P4VRM4 | **√** | **√** | 729 | Putative 26s proteasome regulatory complex subunit rpn11 | A0A0P4VT87 | **√** | **√** |
| 126 | Putative aldehyde dehydrogenase | A0A0P4VJD2 | **√** | **√** | 730 | Putative proteasome-associated protein ecm29 | A0A0N7Z9K2 | **√** | **√** |
| 127 | Protoporphyrinogen oxidase | A0A0P4VSK0 | **√** | **√** | 731 | Putative muscle ras oncoprotein | A0A0P4VPV9 | **√** | **√** |
| 128 | Putative phosphoinositide 3-kinase | A0A0V0G479 | **√** | **√** | 732 | Uncharacterized protein | T1HD78 | **√** | **√** |
| 129 | Putative ribosomal protein l7e | R4G4Q7 | **√** | **√** | 733 | Putative glutamyl-trna synthetase | A0A0N7Z982 | **√** | **√** |
| 130 | Uncharacterized protein | R4G7P2 | **√** | **√** | 734 | Putative short chain dehydrogenase | R4FLI6 | **√** | **√** |
| 131 | Histone H2B | A0A023F5U9 | **√** | **√** | 735 | Uncharacterized protein | T1IAS2 | **√** | **√** |
| 132 | Uncharacterized protein | T1HLW6 | **√** | **√** | 736 | NADH dehydrogenase [ubiquinone] 1 alpha subcomplex subunit 8 | A0A0P4VS72 | **√** | **√** |
| 133 | Putative ribosomal protein l3e | R4G8A6 | **√** | **√** | 737 | Putative vesicle coat complex copii subunit sfb3 | A0A0P4VM67 | **√** | **√** |
| 134 | Putative mitochondrial-processing peptidase subunit beta | A0A0P4VUN1 | **√** | **√** | 738 | Putative fascin-like domain protein | A0A069DZ67 | **√** | **√** |
| 135 | Transgelin | R4FN05 | **√** | **√** | 739 | Methylthioribose-1-phosphate isomerase | A0A0P4VZW9 | **√** | **√** |
| 136 | Histone H2A | A0A023F9G7 | **√** | **√** | 740 | Putative triabin-like lipocalin | R4FPQ1 | **√** | **√** |
| 137 | Putative 60s ribosomal protein l10 | A0A0P4VX82 | **√** | **√** | 741 | Putative nitrophorin | R4G8N0 | **√** | **√** |
| 138 | Glyceraldehyde-3-phosphate dehydrogenase | R4G8R0 | **√** | **√** | 742 | Putative triabin length | R4G560 | **√** | **√** |
| 139 | Spectrin beta chain | A0A0P4VUS4 | **√** | **√** | 743 | Putative triabin length | A0A0P4VJD8 | **√** | **√** |
| 140 | Nitric oxide synthase, salivary gland | Q26240 | **√** | **√** | 744 | Triabin-like lipocalin 3 | Q7YT04 | **√** | **√** |
| 141 | Putative nadh-ubiquinone oxidoreductase ndufs1/75 kDa subunit | A0A0P4W3D4 | **√** | **√** | 745 | Putative lipocalin ai-7 | R4FLF8 | **√** | **√** |
| 142 | Adenosylhomocysteinase | R4G5K4 | **√** | **√** | 746 | Metalloendopeptidase | T1I1C5 | **√** | **√** |
| 143 | Salivary protein MYS3 | Q7YSZ0 | **√** | **√** | 747 | Putative triabin length | R4FQL0 | **√** | **√** |
| 144 | Putative chaperonin | A0A0P4W403 | **√** | **√** | 748 | Putative nitrophorin | A0A0N7Z976 | **√** | **√** |
| 145 | Putative nuclear receptor coactivator 5 | A0A0P4VRC2 | **√** | **√** | 749 | Putative lipocalin ai-7 | A0A0N7Z954 | **√** |  |
| 146 | Putative cytochrome b-c1 complex subunit 2 mitochondrial | A0A0P4VUS2 | **√** | **√** | 750 | Putative salivary protein mys1 | Q7YSZ2 | **√** |  |
| 147 | Histone H4 | A0A023F4Z5 | **√** | **√** | 751 | Putative nitrophorin | A0A0P4VNY0 | **√** |  |
| 148 | Putative chaperonin 10 kd subunit | A0A023FAK4 | **√** | **√** | 752 | Putative lipocalin | R4G4E6 | **√** |  |
| 149 | ATP synthase subunit gamma | A0A0P4VV79 | **√** | **√** | 753 | Lipocalin AI-3 | Q7YT10 | **√** |  |
| 150 | Uncharacterized protein | T1HFF6 | **√** | **√** | 754 | Putative triabin-like lipocalin | A0A0P4W353 | **√** |  |
| 151 | Uncharacterized protein | T1I1Y9 | **√** | **√** | 755 | Putative triabin length | R4FN02 | **√** |  |
| 152 | Uncharacterized protein | T1HTC8 | **√** | **√** | 756 | Putative pallidipin-like lipocalin | R4G426 | **√** |  |
| 153 | ATP-citrate synthase | A0A0P4W2W1 | **√** | **√** | 757 | Heme-binding protein | Q8T5U0 | **√** |  |
| 154 | 60S acidic ribosomal protein P0 | A0A0P4VIK0 | **√** | **√** | 758 | Putative lipocalin ai-7 lipocalin | A0A0P4VNM2 | **√** |  |
| 155 | Putative formyltetrahydrofolate dehydrogenase | A0A0P4VJY8 | **√** | **√** | 759 | Putative lipocalin ai-7 | A0A0P4VJV9 | **√** |  |
| 156 | Putative prohibitins and stomatins of the pid superfamily protein | A0A0P4VHG5 | **√** | **√** | 760 | Putative nitrophorin 3-like protein | A0A0N7Z8Y4 | **√** |  |
| 157 | Protein required for fusion of vesicles in vesicular transport | R4FQ58 | **√** | **√** | 761 | Uncharacterized protein | T1H8M2 | **√** |  |
| 158 | Putative ribosomal protein s2 | R4G8Z8 | **√** | **√** | 762 | Putative nitrophorin 1-like | R4G8M9 | **√** |  |
| 159 | Heterogeneous nuclear ribonucleoprotein k isoform | A0A170ZXR1 | **√** | **√** | 763 | Salivary inositol polyphosphate 5-phosphatase | Q95041 | **√** |  |
| 160 | Putative methylmalonate semialdehyde dehydrogenase | A0A0P4VS52 | **√** | **√** | 764 | Putative lipocalin | R4FR52 | **√** |  |
| 161 | Putative pyridoxal/pyridoxine/pyridoxamine kinase | A0A0N7Z9J2 | **√** | **√** | 765 | Putative triabin-like lipocalin | R4G3G4 | **√** |  |
| 162 | Putative tropomyosin 1 isoform a | A0A069DYF0 | **√** | **√** | 766 | Uncharacterized protein | T1IB91 | **√** |  |
| 163 | Putative nuclear envelope protein | A0A0P4VV21 | **√** | **√** | 767 | Putative nitrophorin 1-like | A0A0P4VRM1 | **√** |  |
| 164 | 60S ribosomal protein L13 | A0A0P4VIY2 | **√** | **√** | 768 | Uncharacterized protein | T1I9F9 | **√** |  |
| 165 | Putative 3-ketoacyl-coa thiolase | A0A0P4VKN4 | **√** | **√** | 769 | Putative lipocalin | R4FLY6 | **√** |  |
| 166 | Putative 2-oxoglutarate dehydrogenase e1 subunit | A0A0P4VSX8 | **√** | **√** | 770 | Uncharacterized protein | T1HHF3 | **√** |  |
| 167 | Superoxide dismutase | R4FJZ0 | **√** | **√** | 771 | Salivary platelet aggregation inhibitor 2 | Q94732 | **√** |  |
| 168 | Dolichyl-diphosphooligosaccharide--protein glycosyltransferase 48 kDa subunit | R4FP71 | **√** | **√** | 772 | Nitrophorin-7 | Q6PQK2 | **√** |  |
| 169 | Putative atp synthase oligomycin sensitivity conferral protein | R4G4K0 | **√** | **√** | 773 | Putative triabin-like protein | R4G3G0 | **√** |  |
| 170 | Uncharacterized protein | T1HG | **√** | **√** | 774 | Ferritin | T1HYY6 | **√** |  |
| 171 | Uncharacterized protein | T1IAC0 | **√** | **√** | 775 | Putative nitrophorin 4b | R4G4L0 | **√** |  |
| 172 | Uncharacterized protein | T1HKS5 | **√** | **√** | 776 | Putative lipocalin | R4FN70 | **√** |  |
| 173 | Putative elongation factor 1 beta | R4G5C0 | **√** | **√** | 777 | Putative lipocalin | R4G530 | **√** |  |
| 174 | Putative vesicle coat complex copi gamma subunit | A0A0P4VNC4 | **√** | **√** | 778 | Putative phosphatidylinositol transfer protein sec14 | R4FJV0 | **√** |  |
| 175 | Putative tropomyosin | A0A0P4VRX2 | **√** | **√** | 779 | Putative triabin length | R4G4H9 | **√** |  |
| 176 | Putative 40s ribosomal protein s13 | A0A023F9K2 | **√** | **√** | 780 | Uncharacterized protein | T1HGK7 | **√** |  |
| 177 | 40s ribosomal protein s14 | A0A023F9M9 | **√** | **√** | 781 | Uncharacterized protein | T1I875 | **√** |  |
| 178 | Putative ca2+-binding actin-bundling protein | A0A069DXE0 | **√** | **√** | 782 | Uncharacterized protein | T1HES3 | **√** |  |
| 179 | Putative ubiquitin-like modifier-activating enzyme 1 | A0A0P4VU82 | **√** | **√** | 783 | Putative lipocalin | R4FPS6 | **√** |  |
| 180 | 40S ribosomal protein S4 | A0A0P4VRS9 | **√** | **√** | 784 | Putative triabin-like lipocalin 2 | A0A0P4VNG9 | **√** |  |
| 181 | Putative 4-aminobutyrate aminotransferase | A0A0P4VZ80 | **√** | **√** | 785 | Putative triabin-like lipocalin | R4FPJ5 | **√** |  |
| 182 | Putative porphobilinogen deaminase | A0A0P4VJZ0 | **√** | **√** | 786 | Uncharacterized protein | T1HDN2 | **√** |  |
| 183 | Putative glycine--trna ligase | A0A0P4VN66 | **√** | **√** | 787 | Putative prokaryotic long-chain fatty acid coa synthetase | A0A0P4VMK1 | **√** |  |
| 184 | Putative s9e ribosomal protein | R4G4I6 | **√** | **√** | 788 | Putative cytoskeletal protein adducin | R4G613 | **√** |  |
| 185 | 40S ribosomal protein S6 | A0A0P4VSS7 | **√** | **√** | 789 | Putative salivary platelet aggregation inhibitor 1 | R4FMZ8 | **√** |  |
| 186 | Putative polyprenyl synthetase | A0A0P4VIE8 | **√** | **√** | 790 | Putative cathepsin l | R4G4T0 | **√** |  |
| 187 | Putative 60s ribosomal protein l26 | A0A0P4VUI5 | **√** | **√** | 791 | Putative sulfotransferase | A0A0P4VP30 | **√** |  |
| 188 | Protein disulfide isomerase | R4G840 | **√** | **√** | 792 | Glutamine synthetase | R4G5A5 | **√** |  |
| 189 | Superoxide dismutase [Cu-Zn] | A0A0P4VTN9 | **√** | **√** | 793 | Uncharacterized protein | T1HW12 | **√** |  |
| 190 | Putative chaperonin subunit 6a zeta | R4G4V8 | **√** | **√** | 794 | Uncharacterized protein | T1H8T1 | **√** |  |
| 191 | Putative sulfotransferase | A0A0P4VL90 | **√** | **√** | 795 | Triabin-like lipocalin 2 | Q7YT05 | **√** |  |
| 192 | Putative s7e ribosomal protein | R4FLZ3 | **√** | **√** | 796 | Uncharacterized protein | T1H8Z9 | **√** |  |
| 193 | Uncharacterized protein | T1H7W9 | **√** | **√** | 797 | Uncharacterized protein | T1H8N3 | **√** |  |
| 194 | Putative 60s ribosomal protein l18 | A0A0P4VRD2 | **√** | **√** | 798 | Uncharacterized protein | T1I074 | **√** |  |
| 195 | Transgelin | A0A0P4VTZ7 | **√** | **√** | 799 | Putative neural cell adhesion molecule 2-like protein | A0A0P4VPG5 | **√** |  |
| 196 | Putative chaperonin | A0A0P4W112 | **√** | **√** | 800 | Cytochrome b-c1 complex subunit Rieske, mitochondrial | A0A0P4VS66 | **√** |  |
| 197 | Uncharacterized protein | T1HLE3 | **√** | **√** | 801 | Putative splicing factor hnrnp-f | A0A0P4VUQ8 | **√** |  |
| 198 | Rab GDP dissociation inhibitor | A0A0P4VKM6 | **√** | **√** | 802 | Uncharacterized protein | T1HAR7 | **√** |  |
| 199 | Putative aminopeptidase npepl1 | A0A0N7Z927 | **√** | **√** | 803 | 6-phosphogluconate dehydrogenase, decarboxylating | A0A0P4VQZ8 | **√** |  |
| 200 | 40s ribosomal protein s15 | A0A161TJK6 | **√** | **√** | 804 | Uncharacterized protein | T1IGI2 | **√** |  |
| 201 | Putative fumarylacetoacetate hydralase | R4G448 | **√** | **√** | 805 | Uncharacterized protein | T1H8H2 | **√** |  |
| 202 | Putative 60s ribosomal protein l13a | A0A0P4VT49 | **√** | **√** | 806 | Translocon-associated protein subunit alpha | A0A170Y3V2 | **√** |  |
| 203 | Putative alkyl hydroperoxide reductase | R4G2X2 | **√** | **√** | 807 | Putative ubiquitin carboxyl-terminal hydrolase 7 | A0A023F463 | **√** |  |
| 204 | Putative gtp-binding adp-ribosylation factor-like protein arl1 | A0A069DPJ5 | **√** | **√** | 808 | Kinesin-like protein | A0A023F5N3 | **√** |  |
| 205 | Putative lysophosphatidic acid acyltransferase lpaat | R4FP29 | **√** | **√** | 809 | Putative nucleotide excision repair factor nef2 rad23 component | A0A069DYN4 | **√** |  |
| 206 | Putative gamma-glutamyl hydrolase | A0A0P4VNM6 | **√** | **√** | 810 | Carboxylic ester hydrolase | T1H8Y3 | **√** |  |
| 207 | Putative fumarylacetoacetase | A0A0N7Z961 | **√** | **√** | 811 | Putative nitrophorin | R4G8N2 | **√** |  |
| 208 | Putative small heat shock protein hsp20 family | A0A0P4VVM9 | **√** | **√** | 812 | Putative triabin-like lipocalin | R4FJ72 | **√** |  |
| 209 | Putative gamma-interferon-inducible lysosomal thiol reductase | R4G4A3 | **√** | **√** | 813 | Putative secreted protein | A0A0P4VL09 | **√** |  |
| 210 | 60S ribosomal protein L18a | R4G4G5 | **√** | **√** | 814 | Uncharacterized protein | T1IFK0 | **√** |  |
| 211 | Putative actin-depolymerizing factor 1 | A0A023F9W1 | **√** | **√** | 815 | Triosephosphate isomerase | A0A0V0G480 | **√** |  |
| 212 | Putative 40s ribosomal protein s16 | R4G3Z7 | **√** | **√** | 816 | Putative trypsin-like protease | A0A0P4VP78 | **√** |  |
| 213 | Putative ribosomal protein l22 | R4FLK0 | **√** | **√** | 817 | Putative nitrophorin 3-like protein | R4G8L8 | **√** |  |
| 214 | Putative endoplasmic reticulum resident protein 44 | A0A0P4VTS4 | **√** | **√** | 818 | Putative cytochrome b5 | A0A0P4VIW4 | **√** |  |
| 215 | Uncharacterized protein | T1I6J7 | **√** | **√** | 819 | Phosphomannomutase | A0A0P4VUS9 | **√** |  |
| 216 | Uncharacterized protein | T1IAP9 | **√** | **√** | 820 | Putative spermidine synthase | A0A0N7Z9A0 | **√** |  |
| 217 | Annexin | T1HHJ6 | **√** | **√** | 821 | Uncharacterized protein | T1HS80 | **√** |  |
| 218 | Putative ca2+-binding actin-bundling protein | A0A0N7Z8Z1 | **√** | **√** | 822 | Putative nitrophorin | R4G800 | **√** |  |
| 219 | Uncharacterized protein | T1HXG5 | **√** | **√** | 823 | Signal recognition particle 54 kDa protein GN=SRP54 | A0A023F5T9 | **√** |  |
| 220 | Eukaryotic translation initiation factor 3 subunit A | A0A0P4W0Q0 | **√** | **√** | 824 | Putative ly-6/neurotoxin superfamily member 1 | A0A0P4VKI5 | **√** |  |
| 221 | Putative ribosomal protein l6 | A0A0P4W283 | **√** | **√** | 825 | Putative salivary protein mys1 | A0A0P4VQA6 | **√** |  |
| 222 | Putative thymosin beta actin-binding motif protein | R4FMW2 | **√** | **√** | 826 | Putative carbonic anhydrase | A0A0P4W0C2 | **√** |  |
| 223 | Uncharacterized protein | T1HTN2 | **√** | **√** | 827 | Putative phosphoglucomutase | A0A0P4VQ68 | **√** |  |
| 224 | Putative sulfotransferase | A0A0P4VND5 | **√** | **√** | 828 | Putative glycosyl transferase family 8 | A0A0P4VPF0 | **√** |  |
| 225 | Putative 40s ribosomal protein s5 | A0A0P4VJL3 | **√** | **√** | 829 | Putative trna and rrna cytosine-c5-methylase | R4G8B4 | **√** |  |
| 226 | Succinate--CoA ligase [ADP/GDP-forming] subunit alpha, mitochondrial | A0A0P4VP23 | **√** | **√** | 830 | Putative alanine--glyoxylate aminotransferase 2 mitochondrial-like isoform x2 | A0A0P4VHK7 | **√** |  |
| 227 | Peptidyl-prolyl cis-trans isomerase | T1I9G6 | **√** | **√** | 831 | Putative translation initiation inhibitor | A0A0P4VJC4 | **√** |  |
| 228 | Ribokinase | A0A0P4VJH2 | **√** | **√** | 832 | Putative triabin-like protein | A0A0P4VSW8 | **√** |  |
| 229 | Glutatione S-transferase, sigma class (GSTs6) | T1IG50 | **√** | **√** | 833 | Putative programmed cell death protein | A0A0P4VSN6 | **√** |  |
| 230 | Putative aspartyl protease | R4FJC3 | **√** | **√** | 834 | Hrp65 isoform x2 | A0A161MBR5 | **√** |  |
| 231 | Putative rna-binding protein musashi/mrna cleavage and polyadenylation factor i complex | R4G3V0 | **√** | **√** | 835 | Uncharacterized protein | T1H7T1 | **√** |  |
| 232 | Putative vesicle coat complex copi beta subunit | A0A0P4W425 | **√** | **√** | 836 | Putative 26s proteasome regulatory complex subunit rpn8/psmd7 | A0A0P4VQ04 | **√** |  |
| 233 | Putative vesicle coat complex copi beta' subunit | A0A0P4VV12 | **√** | **√** | 837 | Uncharacterized protein | T1I1B3 | **√** |  |
| 234 | 40s ribosomal protein s10 | A0A023F9V6 | **√** | **√** | 838 | Putative translation initiation factor 4f ribosome/mrna-bridging subunit eif-4g | A0A023FA61 | **√** |  |
| 235 | Uncharacterized protein | T1IDK1 | **√** | **√** | 839 | Uncharacterized protein | T1HI25 | **√** |  |
| 236 | Uncharacterized protein | T1I611 | **√** | **√** | 840 | Pyruvate dehydrogenase E1 component subunit alpha | A0A0P4VQK4 | **√** |  |
| 237 | Putative 60s ribosomal protein l23 | A0A069DW00 | **√** | **√** | 841 | Putative dolichyl-phosphate-mannose--protein o-mannosyl transferase | R4G3C4 | **√** |  |
| 238 | Putative fumarase | A0A0P4VRQ8 | **√** | **√** | 842 | Putative sec61 protein translocation complex beta subunit | A0A0N7Z990 | **√** |  |
| 239 | Putative synaptobrevin/vamp-like protein sec22 | A0A0P4W2M9 | **√** | **√** | 843 | Putative gamma-glutamyltransferase | A0A0N7Z8Z9 | **√** |  |
| 240 | Putative microsomal signal peptidase 25 kDa subunit spc25 | R4G567 | **√** | **√** | 844 | Uncharacterized protein | T1I462 | **√** |  |
| 241 | Putative troponin t skeletal muscle | A0A0P4VRV0 | **√** | **√** | 845 | Putative acyl-coa synthetase | A0A0P4VJM5 | **√** |  |
| 242 | Putative nadp-dependent isocitrate dehydrogenase | A0A0N7Z8U3 | **√** | **√** | 846 | Putative cathepsin l-like cysteine proteinase | R4FQ93 | **√** |  |
| 243 | Putative short-chain alcohol dehydrogenase/3-hydroxyacyl-coa dehydrogenase | A0A0V0G444 | **√** | **√** | 847 | Calcium-transporting ATPase | A0A0P4VPR1 | **√** |  |
| 244 | Profilin | A0A0P4VU02 | **√** | **√** | 848 | Uncharacterized protein | A0A0N7Z9K7 | **√** |  |
| 245 | Putative purine nucleoside phosphorylase | A0A0P4VNG0 | **√** | **√** | 849 | Putative sodium/potassium-transporting atpase subunit beta-2 | A0A0N7Z8X5 | **√** |  |
| 246 | Putative vesicle coat complex copii subunit sec23 | A0A0P4VMN1 | **√** | **√** | 850 | Translocon-associated protein subunit beta | R4G4P0 | **√** |  |
| 247 | 40S ribosomal protein S15/S22 | A6YPD1 | **√** | **√** | 851 | Mitochondrial thioredoxin 2 | A0A161N1J1 | **√** |  |
| 248 | Putative 60s ribosomal protein l11 | A0A069DQE7 | **√** | **√** | 852 | Putative prokaryotic long-chain fatty acid coa synthetase | A0A0N7Z8U2 | **√** |  |
| 249 | Succinate dehydrogenase (quinone) | A0A0P4VIP5 | **√** | **√** | 853 | Uncharacterized protein | T1I8U8 | **√** |  |
| 250 | Ribosomal protein L14-like protein | G1K056 | **√** | **√** | 854 | Putative triabin-like lipocalin lipocalin | A0A0P4VVH0 | **√** |  |
| 251 | Uncharacterized protein | T1I645 | **√** | **√** | 855 | Putative actin-like protein 87c | A0A069DYT4 | **√** |  |
| 252 | Uncharacterized protein | T1IDR3 | **√** | **√** | 856 | Uncharacterized protein | T1I4Z4 | **√** |  |
| 253 | Putative multifunctional chaperone 14-3-3 family | A0A023F9I8 | **√** | **√** | 857 | Putative gtp-binding adp-ribosylation factor-like protein arl1 | A0A069DQ31 | **√** |  |
| 254 | Proteasome subunit alpha type | A0A0P4W2F9 | **√** | **√** | 858 | Putative rna-binding protein la | R4G871 | **√** |  |
| 255 | Putative transcriptional regulator dj-1 | R4FM64 | **√** | **√** | 859 | Putative translation initiation factor 2c eif-2c | A0A0P4VWF7 | **√** |  |
| 256 | Proteasome subunit alpha type | A0A0P4VNM7 | **√** | **√** | 860 | Putative lipocalin | A0A0P4VU56 | **√** |  |
| 257 | Putative very long-chain specific acyl-coa dehydrogenase mitochondrial | A0A0P4VJV3 | **√** | **√** | 861 | Putative asparagine synthase glutamine-hydrolyzing | A0A0P4VKA9 | **√** |  |
| 258 | Putative receptor mediating netrin-dependent axon guidance | A0A0V0G851 | **√** | **√** | 862 | Uncharacterized protein | T1HPQ5 | **√** |  |
| 259 | Putative vesicle coat complex copii subunit sec31 | A0A0P4VVF3 | **√** | **√** | 863 | Putative rna-binding protein sam68 | A0A023F2B1 | **√** |  |
| 260 | Putative chaperonin | A0A0P4VQV5 | **√** | **√** | 864 | Aminopeptidase | A0A0N7Z8X1 | **√** |  |
| 261 | Inosine-5'-monophosphate dehydrogenase | A0A0P4VV19 | **√** | **√** | 865 | Uncharacterized protein | T1HBQ2 | **√** |  |
| 262 | ATP synthase subunit d, mitochondrial | A0A0P4VTX2 | **√** | **√** | 866 | Putative scaffold/matrix specific factor hnrnp-u/saf-a | A0A0P4VUK4 | **√** |  |
| 263 | Putative dihydrolipoamide succinyltransferase 2-oxoglutarate dehydrogenase e2 subunit | A0A0P4VM99 | **√** | **√** | 867 | Putative cytosol aminopeptidase | A0A0P4VWQ3 | **√** |  |
| 264 | Putative electron transfer flavoprotein ubiquinone oxidoreductase | A0A0P4VSJ0 | **√** | **√** | 868 | Putative enoyl-coa hydratase | R4G3H5 | **√** |  |
| 265 | Coatomer subunit alpha | A0A0P4VMM2 | **√** | **√** | 869 | Cytochrome P450 (CYP6AI) | T1H840 | **√** |  |
| 266 | Malate dehydrogenase | A0A0P4VKA2 | **√** | **√** | 870 | Uncharacterized protein | T1IET8 | **√** |  |
| 267 | Putative aldo-keto reductase | A0A0P4VS02 | **√** | **√** | 871 | Fatty acyl-CoA reductase | T1HPB8 | **√** |  |
| 268 | Transaldolase | A0A0P4VVA6 | **√** | **√** | 872 | Ubiquitin carboxyl-terminal hydrolase | A0A0P4VYK6 | **√** |  |
| 269 | Aminopeptidase | A0A0P4VXS7 | **√** | **√** | 873 | Putative 3-ketoacyl-coa thiolase mitochondrial isoform x1 | A0A0P4VLL1 | **√** |  |
| 270 | Putative cytochrome c oxidase subunit va/cox6 | A0A0N7Z9M0 | **√** | **√** | 874 | Putative conserved plasma membrane protein | A0A0P4VS57 | **√** |  |
| 271 | Putative ribosomal protein l10a isoform 9 | R4FN63 | **√** | **√** | 875 | Putative ras-related protein rab-14 | A0A0V0G5U7 | **√** |  |
| 272 | Putative thioredoxin/protein disulfide isomerase | R4G5U7 | **√** | **√** | 876 | Putative atp-dependent zinc metalloprotease yme1 | A0A0P4VNS7 | **√** |  |
| 273 | Uncharacterized protein | T1I0Q3 | **√** | **√** | 877 | Putative mitochondrial dicarboxylate carrier | R4G8E0 | **√** |  |
| 274 | Proteasome subunit beta type | A0A0P4W204 | **√** | **√** | 878 | Malic enzyme | T1I5N5 | **√** |  |
| 275 | Putative low density lipoprotein receptor | A0A0P4VLG8 | **√** | **√** | 879 | Putative pfkb family carbohydrate kinase | A0A0P4W088 | **√** |  |
| 276 | Histone H3 | A0A069DP67 | **√** | **√** | 880 | Putative bisphosphate 3'-nucleotidase bpnt1/inositol polyphosphate 1-phosphatase | A0A0P4VPR5 | **√** |  |
| 277 | Putative 26s proteasome regulatory complex | R4G8S2 | **√** | **√** | 881 | Putative vesicle coat complex copi zeta subunit | A0A0P4VTE4 | **√** |  |
| 278 | Putative serine/threonine-protein kinase kinx | A0A0P4VP63 | **√** | **√** | 882 | Putative dihydroorotase | A0A0P4VTB4 | **√** |  |
| 279 | Protein disulfide-isomerase a4 | A0A0P4VK18 | **√** | **√** | 883 | Putative salivary nitrophorin 7 | A0A0P4VMA9 | **√** |  |
| 280 | Putative peroxisomal 3-ketoacyl-coa-thiolase | A0A0P4VMS3 | **√** | **√** | 884 | Putative 20s proteasome regulatory subunit beta type psmb1/pre7 | A0A0P4VNN7 | **√** |  |
| 281 | Putative ribosomal protein l23ae | A0A0P4VSW3 | **√** | **√** | 885 | Uncharacterized protein | T1I9V7 | **√** |  |
| 282 | Putative 60s ribosomal protein l21 | A0A0P4VT67 | **√** | **√** | 886 | Putative zn2+-dependent endopeptidase insulinase superfamily protein | A0A0P4VRP5 | **√** |  |
| 283 | Putative emp24/gp25l/p24 family of membrane trafficking protein | R4G4J4 | **√** | **√** | 887 | Putative translation elongation factor ef-1alpha | A0A023F5D9 | **√** |  |
| 284 | Signal recognition particle subunit SRP68 | T1I0Y0 | **√** | **√** | 888 | Putative apoptosis-linked protein-2 | A0A0P4VM48 | **√** |  |
| 285 | Proteasome subunit alpha type | A0A0P4VWN0 | **√** | **√** | 889 | Putative selenoprotein | R4FJH5 | **√** |  |
| 286 | Isocitrate dehydrogenase [NADP] | A0A0P4VZE6 | **√** | **√** | 890 | Putative lysosomal-associated membrane protein | A0A0P4VN45 | **√** |  |
| 287 | Putative vacuolar h+-atpase v1 sector subunit e | A0A0P4VSS8 | **√** | **√** | 891 | Putative adp-ribosylation factor gtpase-activating protein | A0A0P4VU45 | **√** |  |
| 288 | Cytochrome c | A0A161MNS2 | **√** | **√** | 892 | Putative thymidylate synthase | A0A0N7Z948 | **√** |  |
| 289 | Putative rna-binding protein musashi/mrna cleavage and polyadenylation factor i complex | A0A0P4VI20 | **√** | **√** | 893 | Putative mitochondrial tricarboxylate/dicarboxylate carrier | A0A0P4VSU5 | **√** |  |
| 290 | Putative alanyl-trna synthetase | A0A0P4VJS7 | **√** | **√** | 894 | Putative cytochrome c oxidase polypeptide iv | R4FL42 | **√** |  |
| 291 | Signal peptidase complex catalytic subunit SEC11 | A0A0P4VW34 | **√** | **√** | 895 | Putative acetyl-coa carboxylase | A0A0P4VW50 | **√** |  |
| 292 | Putative neoplastic transformation suppressor pdcd4/ma-3 | R4FPP6 | **√** | **√** | 896 | Putative endoplasmic reticulum lectin 1-like isoform x1 | A0A0P4VUT3 | **√** |  |
| 293 | Putative lectin | R4FQE8 | **√** | **√** | 897 | Putative ubiquitin-conjugating enzyme | A0A023F5W1 | **√** |  |
| 294 | Putative ribosomal protein l30 | R4G3Y3 | **√** | **√** | 898 | Putative cytochrome p450 4c1-like protein | A0A0P4W099 | **√** |  |
| 295 | Putative vesicle coat complex copii | R4G581 | **√** | **√** | 899 | Putative nuclear transport factor 2 | A0A0P4VRS7 | **√** |  |
| 296 | Uncharacterized protein | T1HDJ8 | **√** | **√** | 900 | Ferritin | R4G4L4 | **√** |  |
| 297 | Sideroflexin | A0A0N7Z964 | **√** | **√** | 901 | Putative gdp-mannose 46 dehydratase | A0A0P4W3E1 | **√** |  |
| 298 | Putative 20s proteasome regulatory subunit beta type | A0A0N7Z8P5 | **√** | **√** | 902 | Uncharacterized protein | T1HCN4 | **√** |  |
| 299 | Putative 40s ribosomal protein s18 | A0A023F9E5 | **√** | **√** | 903 | Adenylyl cyclase-associated protein | T1I199 | **√** |  |
| 300 | Uncharacterized protein | T1HTR5 | **√** | **√** | 904 | Putative muscle lim protein mlp84b-like isoform 1 | A0A023F8Q4 | **√** |  |
| 301 | Aldehyde dehydrogenase | T1HFT8 | **√** | **√** | 905 | Uncharacterized protein | T1HHQ5 | **√** |  |
| 302 | Multifunctional fusion protein | T1I3T6 | **√** | **√** | 906 | Uncharacterized protein | T1HPJ2 | **√** |  |
| 303 | Dihydrolipoyl dehydrogenase | A0A0N7Z9G7 | **√** | **√** | 907 | Putative translation initiation factor 4f helicase subunit eif-4a | A0A0P4VR40 | **√** |  |
| 304 | Putative 60s ribosomal protein | A0A0P4VRU4 | **√** | **√** | 908 | Lysine--tRNA ligase | A0A0P4VJ03 | **√** |  |
| 305 | Putative transcriptional regulator icp22 | A0A0P4VTT9 | **√** | **√** | 909 | Putative branched chain alpha-keto acid dehydrogenase complex alpha subunit | A0A0N7Z8P6 | **√** |  |
| 306 | S-formylglutathione hydrolase | R4G433 | **√** | **√** | 910 | Putative ubiquinone oxidoreductase ndufa5/b13 subunit | A0A0P4VQ95 | **√** |  |
| 307 | Putative 26s proteasome regulatory complex atpase rpt2 | A0A023FAX2 | **√** | **√** | 911 | Uncharacterized protein | R4G8G9 | **√** |  |
| 308 | Putative translationally controlled tumor protein | A0A0P4VRJ5 | **√** | **√** | 912 | Uncharacterized protein | T1H834 | **√** |  |
| 309 | Putative 26s proteasome regulatory complex subunit | A0A0P4VRL8 | **√** | **√** | 913 | Uncharacterized protein | T1HFD8 | **√** |  |
| 310 | Pyruvate kinase | A0A0P4VNN8 | **√** | **√** | 914 | Putative glutathione s-transferase m2 | A0A0P4VPL2 | **√** |  |
| 311 | Putative 60s ribosomal protein l12 | A0A0P4VKZ5 | **√** | **√** | 915 | Putative signal recognition particle receptor alpha subunit | A0A0P4VUW5 | **√** |  |
| 312 | Putative ribosomal protein l28 | A0A0P4W341 | **√** | **√** | 916 | Putative myosin 2 light chain | A0A0P4VTZ0 | **√** |  |
| 313 | Epoxide hydrolase | R4G4K1 | **√** | **√** | 917 | Putative venom dipeptidyl peptidase 4 | A0A0P4W4J7 | **√** |  |
| 314 | Putative k-similarity type rna binding protein | A0A0P4VT62 | **√** | **√** | 918 | Putative triabin-like lipocalin | R4G8L0 | **√** |  |
| 315 | Putative vesicle coat complex copii subunit sec24/subunit sfb2 | A0A0P4W0W6 | **√** | **√** | 919 | Uncharacterized protein | T1HGN0 | **√** |  |
| 316 | Putative ribosomal protein p2 | A0A0P4VZ88 | **√** | **√** | 920 | Glucose-6-phosphate 1-dehydrogenase | A0A0P4VRU6 | **√** |  |
| 317 | Putative mitochondrial phosphate carrier protein | A0A0P4VZD7 | **√** | **√** | 921 | Putative density-regulated protein | A0A0N7Z8W7 | **√** |  |
| 318 | Uncharacterized protein | T1HC17 | **√** | **√** | 922 | Putative cuticle protein h1c | R4FKS1 | **√** |  |
| 319 | Putative myosin regulatory light chain 2-like protein | A0A0P4VSB9 | **√** | **√** | 923 | Putative cytosolic juvenile hormone binding protein 36 kDa subunit | A0A0P4VQS6 | **√** |  |
| 320 | Uncharacterized protein | T1HTA5 | **√** | **√** | 924 | Putative mrna cleavage factor i subunit | A0A069DPX3 | **√** |  |
| 321 | 40S ribosomal protein S12 | A0A023F9Q2 | **√** | **√** | 925 | Putative 60s ribosomal protein | A0A023F917 | **√** |  |
| 322 | Putative rab subfamily protein of small gtpase | A0A069DXD4 | **√** | **√** | 926 | Uncharacterized protein | A0A0P4VPN1 | **√** |  |
| 323 | Transcription factor BTF3 | A0A023F9P4 | **√** | **√** | 927 | Putative snare protein pep12/vam3/syntaxin 7/syntaxin 17 | A0A0P4VT18 | **√** |  |
| 324 | Putative kynurenine aminotransferase | A0A0P4VTH0 | **√** | **√** | 928 | V-type proton ATPase subunit G | R4G878 | **√** |  |
| 325 | Putative ribosomal protein p1 | A0A0P4VKR9 | **√** | **√** | 929 | Putative mitochondrial solute carrier protein | A0A0P4VNX0 | **√** |  |
| 326 | Adenylate kinase GN=Adk2 | A0A0P4VQJ4 | **√** | **√** | 930 | Putative mitochondrial cytochrome oxidase polypeptide vb | A0A0P4VRL6 | **√** |  |
| 327 | Signal recognition particle subunit SRP72 | R4G5M3 | **√** | **√** | 931 | Uncharacterized protein | T1IDE1 | **√** |  |
| 328 | Uncharacterized protein | T1HRI8 | **√** | **√** | 932 | Putative ubiquinol cytochrome c reductase | A0A0P4VTV8 | **√** |  |
| 329 | Proteasome subunit alpha type | A0A023FCW9 | **√** | **√** | 933 | Putative papain family cysteine protease | R4G361 | **√** |  |
| 330 | Putative 26s proteasome regulatory complex atpase rpt6 | A0A069DU17 | **√** | **√** | 934 | Protein kinase c inhibitor | A0A0P4VVV2 | **√** |  |
| 331 | V-type proton ATPase subunit C | A0A0P4VUX5 | **√** | **√** | 935 | Thymidine phosphorylase | R4G7U4 | **√** |  |
| 332 | Phosphoglycerate mutase | A0A0N7Z9G4 | **√** | **√** | 936 | Eukaryotic translation initiation factor 3 subunit I | A0A069DYL9 | **√** |  |
| 333 | Putative ribosomal s17 | A0A0P4VME1 | **√** | **√** | 937 | 60s ribosomal protein l34-like protein | A0A170XDZ9 | **√** |  |
| 334 | Putative failed axon connections fax protein/glutathione s-transferase-like protein | A0A0P4VZR6 | **√** | **√** | 938 | Putative ubiquitin-40s ribosomal protein s27a-like isoform 1 | A0A069DPN3 | **√** |  |
| 335 | Putative 60s ribosomal protein l17 | A0A023FB83 | **√** | **√** | 939 | Uncharacterized protein | T1I111 | **√** |  |
| 336 | Dihydrolipoamide acetyltransferase component of pyruvate dehydrogenase complex | A0A0N7Z8T0 | **√** | **√** | 940 | Putative cytochrome | A0A0P4VT34 | **√** |  |
| 337 | Putative carnitine o-acyltransferase cpt2/yat1 | A0A0P4VMW2 | **√** | **√** | 941 | Uncharacterized protein | T1H817 | **√** |  |
| 338 | Isocitrate dehydrogenase [NAD] subunit, mitochondrial | A0A0P4VPV8 | **√** | **√** | 942 | Uncharacterized protein | T1I2L4 | **√** |  |
| 339 | Aspartate aminotransferase | A0A0P4VSL5 | **√** | **√** | 943 | Uncharacterized protein | T1I9W8 | **√** |  |
| 340 | Putative ribosomal protein l31e | A0A0P4VZA7 | **√** | **√** | 944 | Putative salivary secreted protein | A0A0P4VZ72 | **√** |  |
| 341 | Vesicle-associated membrane protein-associated protein b | A0A170XJH6 | **√** | **√** | 945 | Putative basement membrane-specific heparan sulfate proteoglycan core protein isoform x13 | A0A0P4VL77 | **√** |  |
| 342 | Uncharacterized protein | T1IBX0 | **√** | **√** | 946 | Putative apoptosis-promoting rna-binding protein tia-1/tiar rrm superfamily | A0A023F4I9 | **√** |  |
| 343 | Putative prohibitin-related membrane protease subunit | A0A0N7Z956 | **√** | **√** | 947 | Putative translation factor | A0A0V0G2X5 | **√** |  |
| 344 | Low-density lipoprotein receptor activity | T1H7T0 | **√** | **√** | 948 | Putative seryl-trna synthetase | A0A0N7Z984 | **√** |  |
| 345 | Uncharacterized protein | T1ICC9 | **√** | **√** | 949 | Putative calponin | A0A0P4VTX0 | **√** |  |
| 346 | S-methyl-5'-thioadenosine phosphorylase | A0A0P4VMV6 | **√** | **√** | 950 | Hipothetical protein | A0A023FB30 | **√** |  |
| 347 | Serine/threonine protein phosphatase 2a regulatory subunit A PE=4 SV=2 | T1H983 | **√** | **√** | 951 | Small ubiquitin-related modifier | A0A023F9R3 | **√** |  |
| 348 | Putative proteasome beta type-2 subunit | A0A0P4VIY8 | **√** | **√** | 952 | Uncharacterized protein | T1IGC5 | **√** |  |
| 349 | Eukaryotic translation initiation factor 3 subunit B | R4G4W6 | **√** | **√** | 953 | Putative dna damage inducible protein | A0A0P4VV02 | **√** |  |
| 350 | Putative rna-binding protein hnrnp-m | A0A0P4W4Z7 | **√** | **√** | 954 | Uncharacterized protein | T1HAI6 | **√** |  |
| 351 | Putative cdk5 regulatory subunit-associated protein 3 | A0A0P4VMZ1 | **√** | **√** | 955 | Putative 26s proteasome regulatory complex subunit rpn2/psmd1 | A0A0P4VTW8 | **√** |  |
| 352 | Malic enzyme | A0A0P4VHP2 | **√** | **√** | 956 | Putative kynurenine aminotransferase glutamine transaminase k | A0A0P4VX02 | **√** |  |
| 353 | Calcium-transporting ATPase | A0A0P4VR76 | **√** | **√** | 957 | V-type proton ATPase subunit | A0A069DSL2 | **√** |  |
| 354 | Putative degradation arginine-rich protein for mis-folding | R4FLG0 | **√** | **√** | 958 | Aconitate hydratase, mitochondrial | A0A069DWY8 | **√** |  |
| 355 | Uncharacterized protein | T1ICI3 | **√** | **√** | 959 | Uncharacterized protein | T1HS06 | **√** |  |
| 356 | Putative alanine aminotransferase | A0A0P4VK84 | **√** | **√** | 960 | Pyrroline-5-carboxylate reductase | A0A0P4VNQ5 | **√** |  |
| 357 | Putative microtubule-associated protein rp/eb family member 1 | A0A0P4VUX6 | **√** | **√** | 961 | NADPH--cytochrome P450 reductase | A0A0P4VTK9 | **√** |  |
| 358 | Uncharacterized protein | T1I4U6 | **√** | **√** | 962 | Putative aspartyl/asparaginyl-trna synthetase translation | A0A0P4VS74 | **√** |  |
| 359 | Uncharacterized protein | T1I012 | **√** | **√** | 963 | Putative eukaryotic translation initiation factor 1a x-chromosomal | A0A069DNX3 | **√** |  |
| 360 | AP complex subunit beta | A0A0P4W4K3 | **√** | **√** | 964 | Eukaryotic translation initiation factor 4E; cap-binding protein | T1IC32 | **√** |  |
| 361 | Putative fibrillarin | A0A0P4VQ22 | **√** | **√** | 965 | Putative hemolymph juvenile hormone binding protein | A0A0N7Z8A2 | **√** |  |
| 362 | Putative 40s ribosomal protein s23 | A0A0V0G2P1 | **√** | **√** | 966 | Uncharacterized protein | T1I7J2 | **√** |  |
| 363 | Cytochrome c oxidase subunit | R4G3W6 | **√** | **√** | 967 | Uncharacterized protein | T1H7Q3 | **√** |  |
| 364 | Putative 26s proteasome regulatory complex | R4G4X6 | **√** | **√** | 968 | Putative collagen alpha chain type iv | A0A069DX02 | **√** |  |
| 365 | Putative medium-chain specific acyl-coa dehydrogenase mitochondrial | A0A0P4VYB9 | **√** | **√** | 969 | Putative basic-leucine zipper transcription factor a | A0A0P4VLS9 | **√** |  |
| 366 | Uncharacterized protein | T1HCC7 | **√** | **√** | 970 | Uncharacterized protein | T1HFD2 | **√** |  |
| 367 | Putative atp-dependent rna helicase | A0A0P4VKG1 | **√** | **√** | 971 | Putative phosphoenolpyruvate carboxykinase | A0A0P4VPA1 | **√** |  |
| 368 | Proteasome subunit alpha type | R4G4N1 | **√** | **√** | 972 | Tyrosine--tRNA ligase | A0A0P4VIM4 | **√** |  |
| 369 | Putative laminin g domain protein | A0A0P4VNA5 | **√** | **√** | 973 | Putative mitochondrial ribosomal protein l50 | A0A0P4VNV2 | **√** |  |
| 370 | alpha-1,2-Mannosidase | A0A0P4W0H0 | **√** | **√** | 974 | Putative n-acetylglucosamine kinase | A0A0P4VT54 | **√** |  |
| 371 | Putative 26s proteasome regulatory complex atpase rpt6 | A0A0N7Z8V3 | **√** | **√** | 975 | Clathrin light chain | A0A069DQ27 | **√** |  |
| 372 | Putative flotillin | A0A0P4VXL4 | **√** | **√** | 976 | Tumor suppressor candidate 3 | A0A170XZ91 | **√** |  |
| 373 | Putative dead-box protein abstrakt | A0A0P4W3R8 | **√** | **√** | 977 | Uncharacterized protein | T1I1X9 | **√** |  |
| 374 | 40S ribosomal protein S24 | A0A0P4VM75 | **√** | **√** | 978 | Uncharacterized protein | T1I640 | **√** |  |
| 375 | Putative leucine-rich acidic nuclear protein | A0A023F8Z9 | **√** | **√** | 979 | Putative mitochondrial/plastidial beta-ketoacyl-acp reductase | A0A0P4VWW1 | **√** |  |
| 376 | Putative transcription factor | A0A069DWV5 | **√** | **√** | 980 | Putative small nuclear ribonucleoprotein e | A0A023FAL6 | **√** |  |
| 377 | Uncharacterized protein | A0A0P4VRF6 | **√** | **√** | 981 | Protein BCCIP homolog | A0A0P4VKT1 | **√** |  |
| 378 | Putative gnl-cdd-187611 cd05353 hydroxyacyl-coa-like dh sdr c-like protein | A0A0P4VTS0 | **√** | **√** | 982 | Putative ubiquitin carboxyl-terminal hydrolase 14 | A0A0N7Z981 | **√** |  |
| 379 | Putative nucleolar protein 56 | A0A0P4VU47 | **√** | **√** | 983 | Putative aquaporin aqpae.a | A0A0P4VU32 | **√** |  |
| 380 | 60S ribosomal protein L36 | A0A0P4W0F3 | **√** | **√** | 984 | Putative chloride intracellular channel exc-4-like protein | A0A023F9J0 | **√** |  |
| 381 | Putative emp24/gp25l/p24 family of membrane trafficking protein | R4G856 | **√** | **√** | 985 | MICOS complex subunit | T1H8J7 | **√** |  |
| 382 | Uncharacterized protein | T1HHI6 | **√** | **√** | 986 | Putative low-density lipoprotein receptor | R4G5R3 | **√** |  |
| 383 | Putative enoyl-coa isomerase | A0A0P4VV62 | **√** | **√** | 987 | Putative nucleosome-binding factor spn pob3 subunit | A0A023FA88 | **√** |  |
| 384 | Putative ubiquinone oxidoreductase ndufa9/39kda subunit | A0A0P4VPK7 | **√** | **√** | 988 | Putative mab-21-like cell fate specification | A0A0P4VPW4 | **√** |  |
| 385 | Putative atp synthase delta chain | A0A0P4VUG0 | **√** | **√** | 989 | Putative olfactory receptor 4-like protein | A0A0P4VW87 | **√** |  |
| 386 | Putative amidase | A0A0N7Z9H6 | **√** | **√** | 990 | Putative oligopeptidase | A0A0P4VSG5 | **√** |  |
| 387 | Putative vesicle trafficking protein sly1 sec1 family | A0A0P4VUA1 | **√** | **√** | 991 | Putative multisynthetase complex | A0A0P4VUL9 | **√** |  |
| 388 | Putative long-chain acyl-coa synthetase amp-forming | A0A0P4W361 | **√** | **√** | 992 | Putative eukaryotic translation initiation factor 3 subunit 12-like protein | A0A0P4VS67 | **√** |  |
| 389 | Uncharacterized protein | T1I6S6 | **√** | **√** | 993 | Putative glyoxylase | R4FNG2 | **√** |  |
| 390 | Putative vacuolar atp synthase subunit h | R4G5L0 | **√** | **√** | 994 | Putative endoplasmic reticulum oxidoreductin-1-like protein | A0A0P4VV13 | **√** |  |
| 391 | Eukaryotic translation initiation factor 5A | A0A069DP22 | **√** | **√** | 995 | Putative carnitine deficiency associated protein | A0A0P4VSQ1 | **√** |  |
| 392 | Putative 26s proteasome regulatory complex subunit rpn3/psmd3 | A0A0P4VL30 | **√** | **√** | 996 | ATP-dependent Clp protease proteolytic subunit | A0A0P4VJM1 | **√** |  |
| 393 | Putative small heat shock protein | A0A0P4VLF4 | **√** | **√** | 997 | Putative thioredoxin and glutathione reductase selenoprotein | A0A0P4VQZ1 | **√** |  |
| 394 | Putative elongation factor 1 delta | R4FLA0 | **√** | **√** | 998 | Putative the pb1 domain found in tfg protein | A0A0P4VV01 | **√** |  |
| 395 | Putative fragile x mental retardation syndrome-related protein 1 | A0A0P4VQ09 | **√** | **√** | 999 | Uncharacterized protein | T1I353 | **√** |  |
| 396 | Uncharacterized protein | T1HS08 | **√** | **√** | 1000 | Uncharacterized protein | T1HXD6 | **√** |  |
| 397 | Putative rab subfamily protein of small gtpase | A0A069DQ39 | **√** | **√** | 1001 | Putative cysteine desulfurase nfs1 | A0A0P4VSF9 | **√** |  |
| 398 | Putative fau protein | A0A0N7Z886 | **√** | **√** | 1002 | Fatty acyl-CoA reductase | R4G4K5 | **√** |  |
| 399 | Putative signal peptidase complex subunit 3-like protein | A0A0N7Z9D8 | **√** | **√** | 1003 | Putative muscle ras oncoprotein | A0A023F5J1 | **√** |  |
| 400 | Putative 40s ribosomal protein s25 | A0A0P4VX92 | **√** | **√** | 1004 | Putative 6-pyruvoyl tetrahydrobiopterin synthase | A0A0P4VQK5 | **√** |  |
| 401 | Putative 60s ribosomal protein l27a | A0A0P4VY84 | **√** | **√** | 1005 | Uncharacterized protein | T1I701 | **√** |  |
| 402 | GrpE protein homolog | A0A0P4VMI1 | **√** | **√** | 1006 | Putative snare protein tlg1/syntaxin 6 | A0A0P4VUQ6 | **√** |  |
| 403 | Pyruvate dehydrogenase E1 component subunit beta | T1IFK5 | **√** | **√** | 1007 | Putative ubiquitin regulatory protein | A0A0P4VW08 | **√** |  |
| 404 | Uncharacterized protein | T1HKY3 | **√** | **√** | 1008 | Uncharacterized protein | T1HWN0 | **√** |  |
| 405 | Putative adaptor protein enigma | R4FQ96 | **√** | **√** | 1009 | Protein required for fusion of vesicles in vesicular transport gamma-snap | A0A0N7Z8N6 | **√** |  |
| 406 | Putative glucosamine 6-phosphate synthetase | A0A0N7Z9G6 | **√** | **√** | 1010 | Uncharacterized protein | T1HDN3 | **√** |  |
| 407 | Tubulin beta chain | A0A069DZ10 | **√** | **√** | 1011 | Putative cytochrome c oxidase | A0A0P4VS79 | **√** |  |
| 408 | Putative hsp70 protein | R4G605 | **√** | **√** | 1012 | Putative replication factor a 1 rfa1 | A0A0P4VM31 | **√** |  |
| 409 | Putative mrna cleavage factor i subunit/cpsf subunit | A0A0P4W057 | **√** | **√** | 1013 | Putative phosphatidic acid-preferring phospholipase a1 | A0A0P4VSW1 | **√** |  |
| 410 | S-(hydroxymethyl)glutathione dehydrogenase | R4G4U8 | **√** | **√** | 1014 | Putative mitochondrial oxoglutarate/malate carrier | A0A0P4VKJ2 | **√** |  |
| 411 | Putative glycerate kinase | A0A0P4VTM8 | **√** | **√** | 1015 | Putative sh3 domain protein sh3glb | A0A023F8U6 | **√** |  |
| 412 | 3-hydroxyisobutyryl-CoA hydrolase, mitochondrial | A0A0P4VVC8 | **√** | **√** | 1016 | Putative 26s protease s4 regulatory subunit | A0A0P4VJE5 | **√** |  |
| 413 | Putative cuticle protein | R4G8D1 | **√** | **√** | 1017 | Putative mitochondrial 28s ribosomal protein s27 | A0A0P4VHF2 | **√** |  |
| 414 | Uncharacterized protein | T1HVU8 | **√** | **√** | 1018 | Putative transducin beta-like 2 | R4G3I1 | **√** |  |
| 415 | Putative rna-binding protein lark isoform x9 | A0A0P4VUU4 | **√** | **√** | 1019 | Putative proteasomal ubiquitin receptor adrm1 | A0A0P4VQL6 | **√** |  |
| 416 | Putative glutathione s-transferase theta-1 | A0A0P4VJZ5 | **√** | **√** | 1020 | Triosephosphate isomerase | A0A0P4VPD0 | **√** |  |
| 417 | GTP-binding nuclear protein | A0A069DQN0 | **√** | **√** | 1021 | Putative elongation factor g mitochondrial | A0A0P4VN27 | **√** |  |
| 418 | Proteasome subunit beta | R4FJF8 | **√** | **√** | 1022 | Putative proteasome inhibitor | A0A0P4VIE1 | **√** |  |
| 419 | Putative nuclear distribution protein nudc | A0A0P4W019 | **√** | **√** | 1023 | Uncharacterized protein | T1HHM1 | **√** |  |
| 420 | Putative eukaryotic translation initiation factor 3 subunit l | A0A0P4VTG6 | **√** | **√** | 1024 | Putative programmed cell death protein | A0A0P4VMD6 | **√** |  |
| 421 | Putative thioredoxin and glutathione reductase selenoprotein | A0A0P4VIW8 | **√** | **√** | 1025 | Putative rab | R4FN87 | **√** |  |
| 422 | Eukaryotic translation initiation factor 3 subunit d | A0A161MJ01 | **√** | **√** | 1026 | Putative zinc transporter | A0A0P4VJL8 | **√** |  |
| 423 | RuvB-like helicase | A0A0P4VJM6 | **√** | **√** | 1027 | Putative conserved secreted protein | A0A0P4VKK0 | **√** |  |
| 424 | Putative cargo transport protein | A0A0P4VUB0 | **√** | **√** | 1028 | Putative apoptosis antagonizing transcription factor/protein transport protein | A0A0P4VH54 | **√** |  |
| 425 | Putative transmembrane protein 14c | R4G7R9 | **√** | **√** | 1029 | Uncharacterized protein | T1IG45 | **√** |  |
| 426 | Putative tropomyosin 1 isoform a | R4FNF3 | **√** | **√** | 1030 | Nuclear pore protein | A0A069DX57 | **√** |  |
| 427 | Putative microtubule-binding protein mip-t3 | R4G8A4 | **√** | **√** | 1031 | Putative caffeine-induced death protein 2 | A0A0P4VT41 | **√** |  |
| 428 | Putative steroid membrane receptor | A0A0N7Z989 | **√** | **√** | 1032 | Ddrgk domain-containing protein 1 | A0A171AIA6 | **√** |  |
| 429 | Acetyltransferase component of pyruvate dehydrogenase complex | A0A0P4VSN4 | **√** | **√** | 1033 | 40s ribosomal protein s19a | A0A171B9K0 | **√** |  |
| 430 | Peptidylprolyl isomerase | A0A0P4VI13 | **√** | **√** | 1034 | Uncharacterized protein | T1HRX6 | **√** |  |
| 431 | Thioredoxin | A0A0P4VTK5 | **√** | **√** | 1035 | Uncharacterized protein | T1HPP2 | **√** |  |
| 432 | Obg-like ATPase 1 | A0A0N7Z9E7 | **√** | **√** | 1036 | Putative mitochondrial aspartate/glutamate carrier protein | A0A0P4VJ93 | **√** |  |
| 433 | Putative glutaminyl-trna synthetase | A0A0P4VXC7 | **√** | **√** | 1037 | Putative myofilin isoform a | A0A0P4VNU1 | **√** |  |
| 434 | Putative threonyl-trna synthetase | A0A0P4VK74 | **√** | **√** | 1038 | Putative glycoprotein glucosyltransferase | A0A0P4VK86 | **√** |  |
| 435 | Proteasome subunit alpha type | A0A0P4VKU2 | **√** | **√** | 1039 | Putative hydroxyacyl-coa dehydrogenase/enoyl-coa hydratase | A0A0N7Z960 | **√** |  |
| 436 | Ubiquitinyl hydrolase 1 | A0A0P4VUD1 | **√** | **√** | 1040 | Uncharacterized protein | T1I4Q0 | **√** |  |
| 437 | Putative endoplasmic reticulum protein erp29 | R4G7T8 | **√** | **√** | 1041 | Putative dynein light chain type 1 | A0A023FAL0 | **√** |  |
| 438 | Putative serine carboxypeptidase s28 | R4G7X4 | **√** | **√** | 1042 | Putative f-actin-capping protein subunit alpha | A0A0P4VN34 | **√** |  |
| 439 | Receptor expression-enhancing protein | A0A0P4VZ98 | **√** | **√** | 1043 | Putative mitochondrial ribosomal protein l17 | A0A0P4VPT3 | **√** |  |
| 440 | Peptidylprolyl isomerase | T1HIE0 | **√** | **√** | 1044 | Arsenical pump-driving ATPase | A0A0P4VRN0 | **√** |  |
| 441 | Putative zn2+-binding dehydrogenase nuclear receptor binding factor-1 | A0A0P4VII7 | **√** | **√** | 1045 | Putative signal recognition particle | A0A0P4VSK9 | **√** |  |
| 442 | Putative a kinase anchor protein | A0A0P4VVW4 | **√** | **√** | 1046 | Putative eukaryotic translation initiation factor 4 gamma 3-like isoform x3 | A0A0P4W4V1 | **√** |  |
| 443 | Uncharacterized protein | R4FQW8 | **√** | **√** | 1047 | Tropomyosin isoform x10 | A0A170Y0V3 | **√** |  |
| 444 | Putative b-cell receptor-associated protein | R4FPZ2 | **√** | **√** | 1048 | Putative dna-bridging protein baf | A0A0P4VRZ0 | **√** |  |
| 445 | Proline dehydrogenase | T1I4L5 | **√** | **√** | 1049 | Putative dnaj-class molecular chaperone | A0A0P4VM44 | **√** |  |
| 446 | Putative mitochondrial aspartate/glutamate carrier protein | A0A0N7Z952 | **√** | **√** | 1050 | Putative eukaryotic translation initiation factor 5 isoform 1 | A0A023F8Y9 | **√** |  |
| 447 | Putative gmp synthase | A0A0N7Z908 | **√** | **√** | 1051 | Uncharacterized protein | T1HIQ8 | **√** |  |
| 448 | Putative atp-dependent rna helicase | A0A069DTC5 | **√** | **√** | 1052 | Tetraspanin | A0A0P4VQ00 | **√** |  |
| 449 | Putative u5 snrnp-specific protein | A0A0V0G7G1 | **√** | **√** | 1053 | Uncharacterized protein | T1HW34 | **√** |  |
| 450 | Uncharacterized protein | A0A0P4VSZ5 | **√** | **√** | 1054 | Uncharacterized protein | T1HT06 | **√** |  |
| 451 | Uncharacterized protein | A0A0P4VK25 | **√** | **√** | 1055 | Putative septins p-loop gtpase | A0A023F9C1 | **√** |  |
| 452 | Putative 26s proteasome regulatory complex subunit | A0A0P4VSA9 | **√** | **√** | 1056 | Putative nucleolar protein nhp2 | A0A0P4VSV5 | **√** |  |
| 453 | Putative programmed cell death protein | A0A0P4VM27 | **√** | **√** | 1057 | Putative lipocalin ai-5 | A0A0V0G8M5 | **√** |  |
| 454 | Ribosomal protein L19 | A0A023FB14 | **√** | **√** | 1058 | Nitrophorin 1A | Q7YT11 | **√** |  |
| 455 | Vesicular integral-membrane protein vip36 | A0A161MMD1 | **√** | **√** | 1059 | Putative nitrophorin | A0A0P4VNY0 | **√** |  |
| 456 | Uncharacterized protein | T1HCY1 | **√** | **√** | 1060 | Putative sulfotransferase | R4G4B4 | **√** |  |
| 457 | Uncharacterized protein | T1HPL5 | **√** | **√** | 1061 | Putative rab subfamily protein of small gtpase | A0A023F9N8 | **√** |  |
| 458 | Uncharacterized protein | T1HB08 | **√** | **√** | 1062 | Putative zn2+-dependent endopeptidase insulinase superfamily protein | A0A0P4VRP5 | **√** |  |
| 459 | Putative rna-binding protein musashi/mrna cleavage and polyadenylation factor i complex | A0A023F2X3 | **√** | **√** | 1063 | Putative translation elongation factor ef-1alpha | A0A0N7Z9I0 | **√** |  |
| 460 | Putative myosin class ii heavy chain | R4FPF0 | **√** | **√** | 1064 | Uncharacterized protein | T1HAE4 | **√** |  |
| 461 | Putative hydroxysteroid dehydrogenase-like protein 2 | A0A0N7Z9I5 | **√** | **√** | 1065 | Putative leucyl-trna synthetase | A0A0P4VR11 | **√** |  |
| 462 | Putative glyoxylase | A0A0P4VMH8 | **√** | **√** | 1066 | Putative glycosyl transferase family | A0A0P4VIC5 | **√** |  |
| 463 | Putative dihydropteridine reductase dhpr/qdpr | A0A0P4VTY0 | **√** | **√** | 1067 | Putative cytochrome c oxidase polypeptide iv | A0A0P4VWS3 | **√** |  |
| 464 | Putative eukaryotic translation initiation factor 3 subunit e | A0A0P4VKZ9 | **√** | **√** | 1068 | Putative trna-binding-domain-containing emap2-like protein | R4G3R9 | **√** |  |
| 465 | Putative ribosomal protein s20 | A0A069DNJ2 | **√** | **√** | 1069 | Putative microtubule-associated protein | A0A0N7Z9J3 | **√** |  |
| 466 | Putative cell wall protein dan4 | A0A0P4VMT0 | **√** | **√** | 1070 | Uncharacterized protein | T1HBW6 | **√** |  |
| 467 | Putative transport protein sec61 alpha subunit | A0A023F5V7 | **√** | **√** | 1071 | Serine/threonine-protein phosphatase | A0A0P4VMT2 | **√** |  |
| 468 | Putative ras-related gtpase | A0A023F9T5 | **√** | **√** | 1072 | Putative membrane coat complex retromer subunit vps35 | A0A069DZJ2 | **√** |  |
| 469 | Putative rab subfamily protein of small gtpase | A0A023F9Y3 | **√** | **√** | 1073 | Putative ubiquitin-activating enzyme e1b | A0A0P4VTI4 | **√** |  |
| 470 | Cytochrome b-c1 complex subunit 7 | A0A0P4VS46 | **√** | **√** | 1074 | Putative camp-dependent protein kinase types i and ii regulatory subunit | A0A023FA94 | **√** |  |
| 471 | Uncharacterized protein | T1HJ42 | **√** | **√** | 1075 | Uncharacterized protein | T1I7P5 | **√** |  |
| 472 | Putative short-chain dehydrogenase | A0A0P4VRY8 | **√** | **√** | 1076 | Putative 5'-3' exoribonuclease 1-like protein | A0A0P4VVA5 | **√** |  |
| 473 | Coatomer subunit epsilon | A0A0P4VNI3 | **√** | **√** | 1077 | Putative aspartyl-trna synthetase | A0A0N7Z950 | **√** |  |
| 474 | 40S ribosomal protein S21 | A0A0P4W0R6 | **√** | **√** | 1078 | Uncharacterized protein | A0A0P4VQX2 | **√** |  |
| 475 | Cytochrome c oxidase subunit 2 | G1K0F0 | **√** | **√** | 1079 | Sorting nexin | A0A069DYW9 | **√** |  |
| 476 | Putative low-density lipoprotein receptor | A0A069DZV0 | **√** | **√** | 1080 | Putative thymidylate kinase/adenylate kinase | A0A0P4VIR8 | **√** |  |
| 477 | Putative ras-related small gtpase rho type | A0A023FBB2 | **√** | **√** | 1081 | Putative 26s proteasome regulatory complex | R4FNT0 | **√** |  |
| 478 | Uncharacterized protein | T1I5N4 | **√** | **√** | 1082 | Putative germ-line stem cell division protein hiwi/piwi | A0A0P4VZ54 | **√** |  |
| 479 | Putative carnitine o-palmitoyltransferase 2 mitochondrial | A0A0P4VNB7 | **√** | **√** | 1083 | Putative hsp90 co-chaperone cdc37 | A0A0P4VUJ1 | **√** |  |
| 480 | Putative translocase of outer mitochondrial membrane complex subunit tom40 | A0A0P4VTG2 | **√** | **√** | 1084 | Putative methylenetetrahydrofolate dehydrogenase/methylenetetrahydrofolate cyclohydrolase | A0A0P4VS87 | **√** |  |
| 481 | 40S ribosomal protein S27 | A0A069DVA7 | **√** | **√** | 1085 | Putative glutathione s-transferase | A0A0P4VLJ6 | **√** |  |
| 482 | Putative rab subfamily protein of small gtpase | A0A023FAU6 | **√** | **√** | 1086 | Putative dehydrogenase with different specificities related to short-chain alcohol dehydrogenase | A0A0P4VGK6 | **√** |  |
| 483 | Putative nadh dehydrogenase subunit e | R4FN97 | **√** | **√** | 1087 | Uncharacterized protein | T1HRQ9 | **√** |  |
| 484 | Putative thiol-disulfide isomerase and thioredoxin | A0A0P4VN33 | **√** | **√** | 1088 | Uncharacterized protein | T1HH37 | **√** |  |
| 485 | RuvB-like helicase | A0A0N7Z9B2 | **√** | **√** | 1089 | Putative aldo/keto reductase family | A0A0P4VRB7 | **√** |  |
| 486 | Putative golgin subfamily protein a member | A0A0P4VW71 | **√** | **√** | 1090 | Proteasome subunit beta type | A0A0P4VT79 | **√** |  |
| 487 | Putative paramyosin | A0A0N7Z8T1 | **√** | **√** | 1091 | Putative selenoprotein | A0A023F970 | **√** |  |
| 488 | Putative isocitrate dehydrogenase alpha subunit | A0A0P4VIG3 | **√** | **√** | 1092 | Putative phenylalanyl-trna synthetase beta subunit | A0A0P4VUN8 | **√** |  |
| 489 | Peptidylprolyl isomerase | T1HDK6 | **√** | **√** | 1093 | D-3-phosphoglycerate dehydrogenase | R4FM92 | **√** |  |
| 490 | Putative cytochrome p450 4c3-like protein | A0A0P4VXN0 | **√** | **√** | 1094 | Putative hect e3 ubiquitin ligase | A0A069DVK8 | **√** |  |
| 491 | Phosphoserine aminotransferase | A0A0N7Z997 | **√** | **√** | 1095 | Putative cytochrome p450 | A0A0P4VNR4 | **√** |  |
| 492 | Putative eukaryotic translation initiation factor 2 subunit 1 | A0A023F987 | **√** | **√** | 1096 | Putative eukaryotic translation initiation factor 2a | A0A0P4VMC4 | **√** |  |
| 493 | 40S ribosomal protein S26 | A0A023F601 | **√** | **√** | 1097 | Putative lipocalin ai-6 | A0A0N7Z8D0 | **√** |  |
| 494 | Putative snare protein pep12/vam3/syntaxin 7/syntaxin 17 | A0A0P4W123 | **√** | **√** | 1098 | Putative lipocalin ai-7 | R4FR66 | **√** |  |
| 495 | Proteasome subunit beta type | A0A0P4VMR1 | **√** | **√** | 1099 | Putative triabin-like lipocalin | A0A0P4VRP3 | **√** |  |
| 496 | Putative molecular chaperone dnaj superfamily protein | A0A0P4VSK2 | **√** | **√** | 1100 | Nitrophorin 3B | Q7YSY5 | **√** |  |
| 497 | Putative pseudouridine synthase | A0A0P4VQH7 | **√** | **√** | 1101 | Putative nitrophorin | A0A0P4VPI4 | **√** |  |
| 498 | Putative secreted protein | A0A0P4VQX3 | **√** | **√** | 1102 | Putative triabin-like lipocalin | A0A0P4VJ86 | **√** |  |
| 499 | Putative ribosomal protein l35e | A0A069DP19 | **√** | **√** | 1103 | Putative nitrophorin | A0A0P4VIM0 | **√** |  |
| 500 | Uncharacterized protein | T1I5U6 | **√** | **√** | 1104 | Putative peroxiredoxin posttranslational modification | A0A0P4VT59 |  | **√** |
| 501 | Uncharacterized protein | T1I8P0 | **√** | **√** | 1105 | Putative two dm9 repeat protein | R4FL58 |  | **√** |
| 502 | Uncharacterized protein | T1I7N2 | **√** | **√** | 1106 | Succinate--CoA ligase [ADP-forming] subunit beta, mitochondrial | A0A0P4VNG2 |  | **√** |
| 503 | Polyadenylate-binding protein | A0A0H2UI64 | **√** | **√** | 1107 | Putative pyrazinamidase/nicotinamidase pnc1 | A0A0V0G4A3 |  | **√** |
| 504 | Putative translocon-associated protein subunit delta | R4FKH5 | **√** | **√** | 1108 | Putative 60s ribosomal protein l44 | A0A069DNX9 |  | **√** |
| 505 | Uncharacterized protein | T1HWP8 | **√** | **√** | 1109 | Putative glutamate/leucine/phenylalanine/valine dehydrogenase | A0A069DVX7 |  | **√** |
| 506 | Lon protease homolog | A0A0P4VU21 | **√** | **√** | 1110 | Uncharacterized protein Rhodnius prolixus | T1HDD0 |  | **√** |
| 507 | Mitochondrial import inner membrane translocase subunit TIM44 | R4FJG7 | **√** | **√** | 1111 | Putative prohibitins and stomatins of the pid superfamily protein | A0A0P4VLH5 |  | **√** |
| 508 | Glutathione peroxidase | R4G379 | **√** | **√** | 1112 | Putative receptor mediating netrin-dependent axon guidance | A0A069DX45 |  | **√** |
| 509 | Palmitoyl-(protein) hydrolase activity | R4FKU8 | **√** | **√** | 1113 | Putative g-protein alpha subunit | A0A0N7Z9B8 |  | **√** |
| 510 | Uncharacterized protein | T1HBI6 | **√** | **√** | 1114 | Uncharacterized protein Rhodnius prolixus | T1HY70 |  | **√** |
| 511 | Uncharacterized protein | T1HVK7 | **√** | **√** | 1115 | Putative mitochondrial ornithine transporter | A0A0P4VSH9 |  | **√** |
| 512 | Putative calmodulin | A0A023F5W8 | **√** | **√** | 1116 | Putative creatine kinase | A0A069DSB2 |  | **√** |
| 513 | Putative serine/threonine kinase | A0A0P4VPI9 | **√** | **√** | 1117 | Putative 15-hydroxyprostaglandin dehydrogenase | R4FM20 |  | **√** |
| 514 | Putative 60s ribosomal protein l24 | A0A023FAE0 | **√** | **√** | 1118 | Alpha-mannosidase | A0A0P4VK91 |  | **√** |
| 515 | NADH-ubiquinone reductase complex 1 | G1K069 | **√** | **√** | 1119 | Putative myosin-2 essential light chain isoform 1 | A0A069DPE1 |  | **√** |
| 516 | Putative phosphoserine phosphatase | R4G3T5 | **√** | **√** | 1120 | Muscle lim protein 1-like protein isoform x8 | A0A170ZG77 |  | **√** |
| 517 | Uncharacterized protein | T1I201 | **√** | **√** | 1121 | Putative c-mpl binding protein | A0A0P4VLH0 |  | **√** |
| 518 | Putative signal recognition particle receptor subunit beta | A0A0P4W032 | **√** | **√** | 1122 | Putative 4-nitrophenylphosphatase domain and non-neuronal snap25-like protein log 1 | R4FM21 |  | **√** |
| 519 | Putative mrna processing protein | A0A0P4VWD4 | **√** | **√** | 1123 | Transferrin Rhodnius prolixus | T1I988 |  | **√** |
| 520 | Uncharacterized protein | T1HYX3 | **√** | **√** | 1124 | Putative golgi protein | A0A0N7Z9K1 |  | **√** |
| 521 | Putative mitochondrial associated endoribonuclease mar1 isochorismatase superfamily | A0A0P4VP13 | **√** | **√** | 1125 | Putative rna-binding protein elav/hu rrm superfamily | A0A023F5T6 |  | **√** |
| 522 | Putative eukaryotic translation initiation factor 3 subunit m | A0A0P4VRL4 | **√** | **√** | 1126 | Cuticle protein-like protein Triatoma matogrossensis | E2J7A8 |  | **√** |
| 523 | Putative alpha-macroglobulin | A0A0P4VGJ9 | **√** | **√** | 1127 | Putative fatty acid oxidation complex | A0A069DWP2 |  | **√** |
| 524 | Putative 17-beta-hydroxysteroid dehydrogenase | A0A0P4VUB5 | **√** | **√** | 1128 | ATP synthase subunit alpha | A0A023F937 |  | **√** |
| 525 | Putative mitochondrial-processing peptidase subunit alpha | A0A0P4VQ80 | **√** | **√** | 1129 | Putative pyruvate dehydrogenase e1 beta subunit | A0A0N7Z9G1 |  | **√** |
| 526 | Putative acyl-coa dehydrogenase | A0A0P4VI52 | **√** | **√** | 1130 | Putative voltage-dependent anion-selective channel isoform 1 | A0A069DRW6 |  | **√** |
| 527 | Uncharacterized protein | T1HHU8 | **√** | **√** | 1131 | Putative glycosylasparagin | R4FJP7 |  | **√** |
| 528 | Putative g-protein beta subunit | A0A023FCL7 | **√** | **√** | 1132 | Putative 60s ribosomal protein l18 | A0A069DQ96 |  | **√** |
| 529 | Putative phenylalanyl-trna synthetase beta subunit | A0A0P4VLW0 | **√** | **√** | 1133 | Uncharacterized protein | A0A0P4VML8 |  | **√** |
| 530 | Putative tomosyn | A0A0P4VYT6 | **√** | **√** | 1134 | 40S ribosomal protein S29 | A6YPJ3 |  | **√** |
| 531 | Tubulin alpha chain | A0A069DUH5 | **√** | **√** | 1135 | Glycerol-3-phosphate dehydrogenase [NAD(+)] | A0A069DSL8 |  | **√** |
| 532 | Putative neural cell adhesion molecule l1 | A0A0P4VF33 | **√** | **√** | 1136 | Putative hydrolase | A0A0P4VGC3 |  | **√** |
| 533 | Putative death-associated protein 1 dap-1 | A0A0P4VUM0 | **√** | **√** | 1137 | Uncharacterized protein Rhodnius prolixus | T1HAV7 |  | **√** |
| 534 | Putative fructose-16-bisphosphatase | A0A0P4VQ33 | **√** | **√** | 1138 | Putative fumarylacetoacetate hydralase | A0A0P4VWK5 |  | **√** |
| 535 | Putative xaa-pro aminopeptidase | A0A0P4VTP9 | **√** | **√** | 1139 | Nucleobindin-2 isoform | A0A161MQF7 |  | **√** |
| 536 | Cchc-type zinc finger protein | A0A023F9L7 | **√** | **√** | 1140 | Putative 3-oxoacyl coa thiolase | A0A069DY |  | **√** |
| 537 | Uncharacterized protein | T1HQI1 | **√** | **√** | 1141 | Uncharacterized protein Rhodnius prolixus | T1HY71 |  | **√** |
| 538 | Putative wd40 repeat stress protein/actin | A0A0P4VZK9 | **√** | **√** | 1142 | Putative von willebrand factor a domain-containing protein 8 | A0A0P4VLT2 |  | **√** |
| 539 | Putative serine/arginine-rich splicing factor 4 | A0A069DYD6 | **√** | **√** | 1143 | Uncharacterized protein Rhodnius prolixus | T1I061 |  | **√** |
| 540 | Putative dehydrogenase with different specificities related to short-chain alcohol dehydrogenase | A0A0P4VRW6 | **√** | **√** | 1144 | Uncharacterized protein | T1HWB5 |  | **√** |
| 541 | Putative mitochondrial atp synthase g subunit | A0A0P4VMW9 | **√** | **√** | 1145 | Putative coproporphyrinogen iii oxidase cpo/hem13 | A0A023FAM8 |  | **√** |
| 542 | Uncharacterized protein | T1HT42 | **√** | **√** | 1146 | Putative apoptosis-linked protein-2 | A0A0V0G5S7 |  | **√** |
| 543 | Putative rrm motif-containing protein | A0A0P4VL88 | **√** | **√** | 1147 | Heterogeneous nuclear ribonucleoprotein h2 isoform | A0A170ZXS3 |  | **√** |
| 544 | Putative molecular chaperone dnaj superfamily | R4G8F2 | **√** | **√** | 1148 | Translocon-associated protein subunit alpha | A0A170Y3R8 |  | **√** |
| 545 | Putative glutaredoxin-related protein | A0A0P4VNB8 | **√** | **√** | 1149 | Alpha-mannosidase | T1HKR4 |  | **√** |
| 546 | Phosphoglycerate kinase | A0A0P4VNT3 | **√** | **√** | 1150 | Putative extracellular matrix glycoprotein laminin subunit beta | A0A023F1J7 |  | **√** |
| 547 | Putative acetyl-coa carboxylase biotin carboxylase subunit | A0A0N7Z931 | **√** | **√** | 1151 | Uncharacterized protein Rhodnius prolixus | T1I985 |  | **√** |
| 548 | Putative cytochrome c1 | A0A0P4VU37 | **√** | **√** | 1152 | Uncharacterized protein | T1IAK6 |  | **√** |
| 549 | MICOS complex subunit MIC13 | A0A0P4VLZ0 | **√** | **√** | 1153 | Putative programmed cell death protein | A0A069DQ13 |  | **√** |
| 550 | Uncharacterized protein | A0A023F2P5 | **√** | **√** | 1154 | Putative mitochondrial associated endoribonuclease mar1 isochorismatase superfamily | A0A069DQG9 |  | **√** |
| 551 | Putative hsp90 co-chaperone p23 | A0A0P4VSE5 | **√** | **√** | 1155 | 60S ribosomal protein L36 | A0A171B7X4 |  | **√** |
| 552 | Proliferating cell nuclear antigen | A0A023F9X0 | **√** | **√** | 1156 | Uncharacterized protein Rhodnius prolixus | T1HN24 |  | **√** |
| 553 | Eukaryotic translation initiation factor 3 subunit H | A0A0P4VS10 | **√** | **√** | 1157 | Putative dna/rna helicase mer3/slh1 dead-box superfamily protein | A0A069DYD2 |  | **√** |
| 554 | Putative 60s ribosomal protein l37 | A0A069DNU8 | **√** | **√** | 1158 | Uncharacterized protein Rhodnius prolixus | T1HV23 |  | **√** |
| 555 | Putative gdp-mannose pyrophosphorylase | A0A0P4VZG6 | **√** | **√** | 1159 | Putative serine/threonine kinase | A0A069DTX7 |  | **√** |
| 556 | Putative vesicle trafficking protein sec1 | A0A0P4VMN7 | **√** | **√** | 1160 | Cytochrome b-c1 complex subunit 7 | R4G412 |  | **√** |
| 557 | Putative heteroproteinous nuclear ribonucleoprotein r rrm superfamily | A0A0P4VZN5 | **√** | **√** | 1161 | Carboxypeptidase | A0A170XJP7 |  | **√** |
| 558 | Putative plasminogen activator inhibitor 1 rna-binding protein | A0A0P4W0H5 | **√** | **√** | 1162 | Uncharacterized protein Rhodnius prolixus | T1I2Z5 |  | **√** |
| 559 | Uncharacterized protein | T1IDK5 | **√** | **√** | 1163 | Putative transferrin-like protein | A0A0P4VVZ2 |  | **√** |
| 560 | Putative phosphoinositide 3-kinase | A0A069DW90 | **√** | **√** | 1164 | Putative kinesin light chain | A0A0N7Z9L6 |  | **√** |
| 561 | Putative cdc42 protein isoform 1 | A0A023F9L3 | **√** | **√** | 1165 | Uncharacterized protein Rhodnius prolixus | T1H9E2 |  | **√** |
| 562 | Putative gamma interferon inducible lysosomal thiol reductase gilt | A0A0P4VUC5 | **√** | **√** | 1166 | Putative transglutaminase/protease-like proteinues | A0A023F227 |  | **√** |
| 563 | Putative actin-related protein arp2/3 complex subunit | A0A0P4VST6 | **√** | **√** | 1167 | Glycerol-3-phosphate dehydrogenase [NAD(+)] | A0A0P4VW60 |  | **√** |
| 564 | Putative cytochrome c oxidase assembly protein | A0A0P4VQS1 | **√** | **√** | 1168 | Branched-chain-amino-acid aminotransferase | A0A0P4VNW2 |  | **√** |
| 565 | Uncharacterized protein | T1I6B2 | **√** | **√** | 1169 | Putative dolichyl-diphosphooligosaccharide--protein glycosyltransferase subunit stt3a | A0A0N7Z980 |  | **√** |
| 566 | Putative short chain acyl-coa dehydrogen | A0A0P4VS76 | **√** | **√** | 1170 | Putative mitochondrial solute carrier protein | A0A0P4VU50 |  | **√** |
| 567 | Putative receptor-like serine/threonine kinase | R4G3H4 | **√** | **√** | 1171 | Putative sorting and assembly machinery component 50 | A0A0P4VMT8 |  | **√** |
| 568 | Putative serine hydrolase | A0A0N7Z8U6 | **√** | **√** | 1172 | Putative dual specificity phosphatase | A0A0P4VU03 |  | **√** |
| 569 | Putative pre-mrna-splicing factor | A0A0P4VU40 | **√** | **√** | 1173 | Putative mrna cleavage factor i subunit | A0A0P4VII3 |  | **√** |
| 570 | Putative glyoxalase | A0A0P4VZF5 | **√** | **√** | 1174 | Putative ras-related protein rab-14 | A0A0P4VRG0 |  | **√** |
| 571 | Putative glutathione s-transferase | A0A0P4VSA5 | **√** | **√** | 1175 | Putative atp-dependent zinc metalloprotease yme1 | A0A069DXI0 |  | **√** |
| 572 | Uroporphyrinogen III synthase | T1HKN9 | **√** | **√** | 1176 | Putative rna-binding protein 45-like isoform x3 | A0A0P4VVQ2 |  | **√** |
| 573 | Putative small nuclear ribonucleoprotein sm d3 | A0A069DPB6 | **√** | **√** | 1177 | Uncharacterized protein Rhodnius prolixus | T1I9C4 |  | **√** |
| 574 | Putative lysosomal alpha-glucosidase-like protein | A0A0P4VP68 | **√** | **√** | 1178 | Putative 26s proteasome regulatory complex subunit | A0A0P4VUT1 |  | **√** |
| 575 | Putative reticulocalbin | R4G8E7 | **√** | **√** | 1179 | Uncharacterized protein | T1IA74 |  | **√** |
| 576 | Putative atp-dependent rna helicase | A0A0P4VYW0 | **√** | **√** | 1180 | Putative nuclear pore complex rnpl4 component sc npl4 | A0A0V0G7N4 |  | **√** |
| 577 | Putative flavin-containing monooxygenase | A0A0P4VNC7 | **√** | **√** | 1181 | Putative arginine/serine-rich splicing factor | A0A023FAD0 |  | **√** |
| 578 | Serine/threonine-protein phosphatase | A0A023F4 | **√** | **√** | 1182 | Uncharacterized protein | T1HP43 |  | **√** |
| 579 | Putative quinone oxidoreductase/flavo-binding protein | A0A0P4VRG6 | **√** | **√** | 1183 | Putative enoyl-coa isomerase | A0A0P4VGM4 |  | **√** |
| 580 | Peptidylprolyl isomerase | A0A0P4VW25 | **√** | **√** | 1184 | Putative mrna processing protein | A0A0P4VWD4 |  | **√** |
| 581 | Putative the phosphoinositide binding phox similarity domain of sorting nexin 3 | A0A069DPS7 | **√** | **√** | 1185 | Serine arginine-rich splicing factor 7-like protein isoform | A0A161MAD8 |  | **√** |
| 582 | Putative troponin i | A0A069DPR6 | **√** | **√** | 1186 | Putative alcohol dehydrogenase class iv | A0A0P4W3B5 |  | **√** |
| 583 | Putative mitochondrial carrier protein mrs3/4 | A0A0P4VUQ9 | **√** | **√** | 1187 | Putative actin-binding lim zn-finger protein limatin involved in axon guidance | A0A0P4VPG9 |  | **√** |
| 584 | Putative flotillin | A0A0N7Z922 | **√** | **√** | 1188 | Dead-box atp-dependent rna helicase 20-like protein isoform | A0A161TF70 |  | **√** |
| 585 | Putative dolichol-phosphate mannosyltransferase | R4FLT6 | **√** | **√** | 1189 | Methionine aminopeptidase 2 | R4G5K9 |  | **√** |
| 586 | Putative ribosomal protein l38 | A0A023FCL4 | **√** | **√** | 1190 | Putative thiamine pyrophosphokinase | A0A0P4VVA0 |  | **√** |
| 587 | Putative basement membrane-specific heparan sulfate proteoglycan core protein | A0A0P4VKE4 | **√** | **√** | 1191 | Putative glutathione s-transferase | A0A023FAZ9 |  | **√** |
| 588 | Uncharacterized protein | A0A0P4VHX7 | **√** | **√** | 1192 | Putative gtp-binding adp-ribosylation factor arf6 darf3 | A0A023F9X4 |  | **√** |
| 589 | Putative thioredoxin peroxidase 1 | A0A0P4VWU5 | **√** | **√** | 1193 | Putative zinc-binding oxidoreductase | A0A0P4VHR1 |  | **√** |
| 590 | Putative membrane protein | A0A0P4VRZ7 | **√** | **√** | 1194 | Putative tfiif-interacting ctd phosphatase | A0A0P4VTB3 |  | **√** |
| 591 | Aminopeptidase | A0A0P4VSA7 | **√** | **√** | 1195 | Putative concentrative na+-nucleoside cotransporter cnt1/cnt2 | A0A0P4VPC0 |  | **√** |
| 592 | Putative enhancer of mrna-decapping protein 4 | A0A0P4VU27 | **√** | **√** | 1196 | Putative ras-related protein rac1 | A0A0P4VSZ1 |  | **√** |
| 593 | Uncharacterized protein | T1HTY7 | **√** | **√** | 1197 | Low-density lipoprotein receptor activity Rhodnius prolixus | T1H8E1 |  | **√** |
| 594 | Putative translation initiation factor eif-2b subunit beta | A0A069DS82 | **√** | **√** | 1198 | Putative bag family molecular chaperone regulator 2 | A0A0P4VT04 |  | **√** |
| 595 | Putative karyopherin importin beta 3 | A0A0P4VQV9 | **√** | **√** | 1199 | Uncharacterized protein Rhodnius prolixus | T1HS09 |  | **√** |
| 596 | Uncharacterized protein | T1I3M9 | **√** | **√** | 1200 | Putative gtp-binding nuclear protein ran1 | A0A0N7Z8T2 |  | **√** |
| 597 | Uncharacterized protein | T1ICF1 | **√** | **√** | 1201 | Putative steroid reductase required for elongation of the very long chain fatty acids | A0A0P4VMW4 |  | **√** |
| 598 | NAD-dependent protein deacylase | A0A0P4VPP7 | **√** | **√** | 1202 | Putative mitochondrial oxodicarboxylate carrier protein | A0A069DRN1 |  | **√** |
| 599 | Serine/threonine-protein phosphatase | A0A023F4Y9 | **√** | **√** | 1203 | Putative endocytosis/signaling protein ehd1 | R4G5H8 |  | **√** |
| 600 | Uncharacterized protein | T1HWS5 | **√** | **√** | 1204 | Coatomer subunit delta | A0A023F8Z5 |  | **√** |
| 601 | Putative cytosolic ca2+-dependent cysteine protease calpain | A0A0N7Z8S7 | **√** | **√** | 1205 | Putative nicotinic acid phosphoribosyltransferase | A0A0N7Z8J2 |  | **√** |
| 602 | Putative ubiquinone oxidoreductase ndufs8/23 kDa subunit | A0A0P4VTL7 | **√** | **√** | 1206 | Putative carbon-nitrogen hydrolase | A0A0P4VNQ6 |  | **√** |
| 603 | Putative hydroxymethylglutaryl-coa lyase | A0A0P4VNQ2 | **√** | **√** | 1207 | Putative hexamerin | A0A0V0GDS7 |  | **√** |
| 604 | Putative nadh-ubiquinone oxidoreductase ndufs3/30 kDa subunit | A0A0P4VHG1 | **√** | **√** | 1208 | Putative lipocalin ai-7 | R4G8K0 |  | **√** |
