# Supplementary material for: Comparative proteomic analysis of the hemolymph and salivary glands of Rhodnius prolixus and R. colombiensis reveals candidates associated with differential lytic activity against Trypanosoma cruzi Dm28c and T. cruzi Y
Source: PLoS Negl Trop Dis. 2024 Apr 3;18(4):e0011452. doi: 10.1371/journal.pntd.0011452 (PMC10990223; doi:10.1371/journal.pntd.0011452)
Supplement: Attachment — Submitted filename: Letter_reponse_PLOS Neglected Tropical Diseases.pdf [file pntd.0011452.s004.pdf]

Ibagué, Colombia

30th January, 2024

Dr. Edmundo Carlos Grisard, BSc, PhD
Guest Editor
PLOS Neglected Tropical Diseases

Dr. Ricardo Fujiwara
Section Editor
PLOS Neglected Tropical Diseases

Dear Dr Carlos Grisard and Dr Ricardo Fujiwara, we would like to thank you for your editorial evaluation and the associated peer review of our manuscript formerly entitled, "Comparative proteomic analysis of *Rhodnius prolixus* and *R. colombiensis* haemolymph and salivary glands has revealed candidates associated with differential lytic activity against *Trypanosoma cruzi* I and *T. cruzi* II". The modifications and corrections to the MS suggested by the evaluators have been made and our replies to the queries raised are attached below; we therefore hope that this new version of the MS now meets your journal's requirements for publication.

Yours sincerely,

Hamilton Julián Barbosa Vinasco

Corresponding author

****

Reviewer's Responses to Questions

Key Review Criteria Required for Acceptance?</br></br>
As you describe the new analyses required for acceptance, please consider the following:</br></br>

Methods</br></br>
-Are the objectives of the study clearly articulated with a clear testable hypothesis stated?</br>
-Is the study design appropriate to address the stated objectives?</br>
-Is the population clearly described and appropriate for the hypothesis being tested?</br>
-Is the sample size sufficient to ensure adequate power to address the hypothesis being tested?</br>
-Were correct statistical analysis used to support conclusions?</br>
-Are there concerns about ethical or regulatory requirements being met?</br></br>

Reviewer #1: (No Response)

Reviewer #2*: The primary objective was to confirm the differential lytic activity in the hemolymph and components of salivary glands of R. prolixus and R. colombiensis against epimastigotes and trypomastigotes of T. cruzi I and T. cruzi II. This cannot be confirmed using only a single strain per DTU*

- *Authors failed to mention the repository where the proteomic data was placed*.

R: The mass spectrometry proteomics data have been deposited to the ProteomeXchange Consortium via the PRIDE partner repository with the dataset identifier PXD048605".

The data will be enabled on the platform once the article published in relation to the data is notified.

username: reviewer_pxd048605@ebi.ac.uk

Password**:** 5UZLHESm

- *Did the authors evaluate the protein integrity in the pepsin treated control hemolymph? (SDS-PAGE gels or otherwise?). It would be of interest if they could specify if they did.*

R: We did not directly assess protein integrity in control hemolymph. We only infer that the fact that it lost its ability to lyse the Y strain parasites, the proteins would have lost their functional properties.

- *Did the control haemolymph also contain phenyl thiourea? Please clarify*

R: Similarly to sample treatment, both negative and positive controls were treated with phenylthiourea (this has been added to line 156).

- *Line 182: "10 µL of T. cruzi culture forms containing a concentration of 2.5–3.5 ×107 parasites/mL" what were these parasites suspended in before mixing 1:1 with LIT?*

R: 10 µL of culture forms of *T. cruzi* suspended in LIT (10% FBS), leaving a final concentration of 2.5–3.5 $\times 10^7$ (added in text, line 192).

- *In line 185: what is 10% LIT? What was the LIT medium diluted in? Or are the authors refering to LIT medium containing 10% FBS? Please clarify*

R: Liver infusion tryptose (LIT) medium contains 10% foetal bovine serum (FBS)

Conclusions</br></br>
-Are the conclusions supported by the data presented?</br>
-Are the limitations of analysis clearly described?</br>
-Do the authors discuss how these data can be helpful to advance our understanding of the topic under study?</br>
-Is public health relevance addressed?</br></br>

Reviewer #1: (No Response)

Reviewer #2: *- T. cruzi is highly variable. Great variability exists within DTUs, specially TcI. Having tested only one strain for each DTU, the authors cannot generalize findings to entire DTUs. The entire manuscript needs to be edited, even figures and figure legends, where labels should read "Dm28c" instead of TcI and "Y" instead of TcII. Currently, the manuscript overgeneralizes conclusions to entire DTUs, which is not supported by the data from a single strain per DTU. Therefore, the conclusions and discussion need to be carefully revised to ensure no overgeneralizations, which extend beyond what is supported by the data, are made.*

R: The title has been modified to read, "Comparative proteomic analysis of *Rhodnius prolixus* and *R. colombiensis* haemolymph and salivary glands has revealed candidates associated with differential lytic activity against *Trypanosoma cruzi* Dmc28c and *T. cruzi* Y". TcI has now been replaced by Dmc28c and TcII by Y throughout the whole text.

- *T. cruzi infects the vector´s digestive tract. Therefore, during infection of triatomines, the parasite is not in contact with haemolymph. As a result, the fact that the haemolymph displays lytic effect against some strains and not others would not affect the completion of the parasite´s life cycle. Therefore, authors need to explain why is it relevant to study the lytic effect of haemolymph as opposed to, for example, proteins expressed in the vector digestive tract.*

R: (added line 583) *Rhodnius prolixus*'s specific lytic activity against the *T. cruzi* Y strain, but not against the *T. cruzi* dm28C strain, is probably the result of the overexpression of genes such as nitrophorins (NPs). These proteins are involved in the metabolism of NO, a free radical that acts in the constitutive innate immunity of the insect (Knipp & He, 2011)…(added line 588) Nitrophorin 4-A (NP4) was found in *R. prolixus* haemolymph in this work; NP4 is important because nitric oxide (NO) from nitrophorins could be released in the foregut and act on the genotypes of parasites which are sensitive to oxidative stress. *In vivo* experiments have shown that increased NO production in *R. prolixus* hemolymph and digestive tract has correlated with decreased in *T. cruzi* multiplication; on the contrary, the parasite manages to maintain and multiply itself when NO production becomes decreased (Batista *et al*., 2020).

(added line 596) Although the mechanism regarding how NPs are expressed in haemolymph remain unknown, it is likely that they are secreted directly into the haemocoel as a factor of humoral immunity (García *et al*., 2010). Other immune factors, such as antimicrobial peptides, reactive nitrogen and oxygen intermediates and complex enzymatic cascades (such as the prophenoloxidase system) contributing to coagulation or melanisation in the haemolymph, also act as immune response components for eliminating potential pathogens acquired during feeding.

.

- *Although the proteomic analysis is interesting, there are no specific experiments connecting the lytic effect in the hemolymph or saliva with specific proteins. The authors only speculate regarding possible factors involved in the lysis, based on the relative abundance of proteins for each vector species.*

R: Candidate molecules must/will be synthesised in *in vitro* experiments to account for the observed lytic activity to verify their trypanolytic activity.

Editorial and Data Presentation Modifications?</br>

Use this section for editorial suggestions as well as relatively minor modifications of existing data that would enhance clarity. If the only modifications needed are minor and/or editorial, you may wish to recommend "Minor Revision" or "Accept".

Reviewer #1: (No Response)

Reviewer #2: (No Response)

Summary and General Comments</br></br>
Use this section to provide overall comments, discuss strengths/weaknesses of the study, novelty, significance, general execution and scholarship. You may also include additional comments for the author, including concerns about dual publication, research ethics, or publication ethics. If requesting major revision, please articulate the new experiments that are needed.

Reviewer #1: *The study by Barbosa and colleagues entitled "Comparative proteomic analysis of the hemolymph and salivary glands of Rhodnius prolixus and R. colombiensis reveals candidates associated with differential lytic activity against Trypanosoma cruzi I and T. cruzi II" evaluated the trypanolytic effect of hemolymph and saliva proteins of R. prolixus and R. colombiensis, using two strains of T. cruzi, Dm28c and Y. The study also described and compared the hemolymph and saliva proteome between the two Rhodnius species. Finally, they tried to associate the immune proteins found in the proteome with the trypanolytic effects observed in the in vitro experiments. The experimental design is sound and the methodology employed is appropriate and well established. However, there are some issues in methodology and results that should be addressed before the manuscript is accepted for publication.*

- *Line 122. Check that the strain used was not Dm28c.*

R: Dm28c (corrected throughout the text)

- *Line 129. Describe in more detail how the triatomines were fed, including information on the ethics of animal use.*

R1: The state of the chickens at feeding time and that of the insects at feeding time have been described (line 133).

R2: A section entitled *Ethical aspects has now been included (in Materials and Methods).*

- *Line 137. When describing the methodology and results, the authors generalized the strains used as TCI and TCII, but only one strain of each DTU was tested. In addition, as Dm28c and Y are reference strains, which have been maintained in culture medium for a long time, they may not be the best representatives of these DTUs. Therefore, authors should replace TCI with Dm28c and TCII with Y throughout the text.*

R1: TcI has now been replaced by DM28c and TcII by Y throughout the text.

R2: The discussion concerning discrete typing units (DTU) and lytic effect has been broadened, based on data reported by Yasmin Suarez *et al*., (2020) whose group used 20 TcI and 2 TcII strains; their research led to similar results to those presented here (added line 702).

- *Line 148. How many biological replicates were used?*

R: Each experiment was done in triplicate (added to line 176).

- *Line 159. Were these parasites obtained from culture medium? Include details of this procedure.*

R: 1x108 Dm28c and Y strain epimastigote/mL were cultured at 28°C in 4 mL liver infusion tryptose (LIT) medium, supplemented with 5% heat-inactivated FBS. The parasites were kept in such conditions for 10 to 12 days; a significant increase in metacyclic trypomastigotes was observed during such time due to nutritional stress. The medium was then passed through a negatively-charged, Sepharose-DEAE (diethylaminoethyl resin) column to retain the epimastgotes and separate them from the metacyclic trypomastigotes (added line 163).

- *Line 168. Were these samples technical or biological replicates?*

R: biological replicas (added line 176)

- Line 181. Why did the authors not use trypomastigote forms for this experiment? These are the forms that will be in contact with the triatomine saliva that is ingested during the feeding process.

R: Although conducting experiments using trypomastigote forms and insect saliva was considered, limitations had to be faced regarding the available biological material. Collecting saliva from a single insect only produces 1-2 μL and the insect must also be sacrificed; the experiment would thus have needed a minimum of 60 insects per species.

- Line 185. Why is the negative control of saliva assays different from that of hemolymph?

R: Our limitation was also the biological material. A relatively good amount of saliva is required to be able to subject the tissue to pepsin and subsequently the pH of the solution must be measured with pH indicator paper and this paper absorbs a good part of the collected tissue. In the case of hemolymph, up to 100μL of tissue per insect can be collected, so it is easier to obtain control with pepsin. In saliva, 1-2 μL of saliva is collected per insect.

- Line 190. Anova is not the correct statistical test to compare these data, which are dependent as they come from the same sample measured at different times. Authors should reanalyze them using a test suitable for repeated samples.

R: We adjusted the design and the very correct observation was taken into account. Our design aims to compare the treatments and controls taking into account that the measurements are repeated, since the same individuals were measured at hour 0 and hour 14 (in hemolymph). To do this, we carry out two steps: 1) a comparison of paired means is made, taking the measurements from hour 0 and hour 14 as pairs for everyone. 2) the differences calculated in the previous step are analyzed using a one-way ANOVA in which the treatments and controls are compared to each other (tukey test). All comparisons were made at 5% significance level.

Line 197. What was the number of samples used? Describe how samples were prepared for LC/MS/MS and the conditions used in the mass spectrometer.

R: (ítem added line 244)

Protein digestion

The proteins were extracted from the gels and plated onto 96-well plates by washing with ultrapure water; protein digestion was carried out according to Shevchenko *et al*.'s protocol (1996), with modifications suggested by Havlis *et al*. (2003). The proteins were reduced in 10 mM DTT and alkylated with 55 mM iodoacetamide (IAA) and digested with 126 nM sequencing grade modified porcine trypsin (Promega Madison, WI) at 37°C overnight. The digestion products were extracted with 1% formic acid in 2% acetonitrile, followed by 1% formic acid and 50% acetonitrile. The extracts were vacuum centrifuged, dried and then suspended in 12 µl 0.1% formic acid. Liquid chromatography with tandem mass spectrometry (LC-MS/MS) was used for analysing 5 µl of the extracts.

LC-MS/MS analysis

Reversed-phase capillary nanoliquid chromatography (nanoLC) was used for separating the peptides in the samples, which were then analysed by electrospray mass spectrometry (ES-MS/MS)

An Agilent 1200 nanopump connected to a 5600 mass spectrometer (AB SCIEX, Framingham, MA, USA), equipped with a nanoelectropulverisation ion source, was used for the experiments. The peptides were separated on a half-packed PicoFrit column (New Objective, Woburn, MA) (3U, 100 A C18, 15 cm x 0.075 mm internal diameter); they were then eluted with a 5-35% linear gradient with dissolvent B (acetonitrile, 0.1% formic acid) for 64 min at 300 nl/min. The mass spectra were obtained using the data-dependent acquisition mode with Analyst software (SCIEX, version 1.7). Each full scan of the mass spectra (400 at 1,250 m/z) was followed by the dissociation induced by the collision of the 20 most intense ions. Dynamic exclusion was carried out for 12 s at 100 ppm tolerance.

- Line 198. If the procedure was the same as the previous experiment, describe the details the first time you mention it.

R: Details given previously. However, the initial experiment was related to a lysis experiment and the second for sequencing, so there may have been some changes, such as adding or not adding phenylthiourea, so we consider it important to mention it in this section.

Line 202. How many salivary glands were used?

R: 20 pairs of glands (added)

-Line 208. Why was hemolymph exposed to a cell lysis procedure if it was previously centrifuged to remove the cells?

R: Although the lysis buffer (40 mm tris-base, 7 m urea, 2 m thiourea, 4% chaps, 1 mm PMSF) is used for cell lysis, also facilitates the denaturation, solubilization and protection of proteins, which It is necessary for post-extraction processes such as SDS-Page and electrophoresis LC/MS/MS.

The PMSF (Phenylmethylsulfonyl fluoride) for example, acts as a protease inhibitor which is very necessary to protect the integrity of proteins for later sequencing processes. CHAPS, acts as a soft, non -ionic detergent, which facilitates the solubilization of proteins, maintaining the integrity of them. Urea and thiourea act as chaotropic agents weakening the hydrophobicity of proteins to solubilize them.

- Line 256. I suggest replacing the title with: Effects of hemolymph from R. prolixus and R. colombiensis on epimastigotes…

R: We accept the change.

Line 263. Metacyclic epimastigotes? I suggest revising the language in the results section.

R: It was corrected. (metacyclic trypomastigote)

- Line 269. Include, at least in the description, the results of the negative and positive controls (same for fig. 2).

R: The result has been mentioned and supplementary material added regarding the parasite counts.

- Line 280. I suggest replacing the title with: Effects of R. prolixus and R. colombiensis salivary gland components on T. cruzi epimastigotes.

R: We accept the change.

- Line 303. In the study by Ouali et al (2022), 376 proteins were identified in the hemolymph of R. prolixus. Include this study on the discussion of these results. Ouali, R., Vieira, L. R., Salmon, D., & Bousbata, S. (2022). Rhodnius prolixus Hemolymph Immuno-Physiology: Deciphering the Systemic Immune Response Triggered by Trypanosoma cruzi Establishment in the Vector Using Quantitative Proteomics. Cells, 11(9), 1449.

R: We carry out a discussion in two segments of the Discussion item:

(line 483) The proteomic results provided our first approach to describing such immune factors. The total amount of proteins identified in this work (120 in *R. prolixus* and 107 in *R. colombiensis*) was less than that reported in other work, such as that by Ouali *et al*., (2022) who mentioned the detection of up to 269 constitutively expressed proteins. We consider that two variables could explain our lowest value regarding the total number of proteins identified: i) our filter for considering that a protein had been detected in the tissue took the number of *peptide sequences unique to a protein group* as being at least 2, whilst the work by Ouali *et al*., (2022) reported proteins having a *unique peptide number* from 1 onwards. *ii*) Our methodology required the haemolymph to be centrifuged to eliminate any cell type. We thus expected that fewer proteins would be detected or at least, only those that are constitutively secreted. 40 proteins related to immunity were the object of interest in our study (Figure 8); this number of proteins was close to that reported by Ouali *et al*., (2022) who mentioned 58, such figure considered that they also included some proteins whose expression is induced.

(line 571). Other research has already identified NPs in *R. prolixus* haemolymph. Ouali *et al*., (2022) analysed haemolymph protein expression regarding *T. cruzi* infection and, although these proteins' role concerning insect immunity was not discussed regarding the objective of their work, it can be seen that 4 NP

identifications were reported in the supplementary material (Q7YT15, Q26239, T1HKP3, R4G8M6). A recent study by Santos *et al*., (2022) on lipocalins (which included nitrophorins) mentioned that such proteins could be present in other types of tissue and could have multiple functions. The authors suggested that central nervous system expressed lipocalins the can act as neurotransmitters, as odorant-binding proteins (OBPs) when expressed in antennae and as lipid transporters when expressed in the haemolymph.

Line 376. I wouldn't generalize the results to DTU genotypes, as only one strain of each DTU was tested.

R: The suggestion has been accepted and pertinent changes have been made throughout the entire document.

Line 444. I cannot understand how a protein that is enclosed in the salivary glands would cross the membrane to reach the hemolymph. In the case they are not synthetized in the hemolymph, could be the presence of these proteins a contamination caused by the rupture of the salivary glands during insect dissection?

R1: Any type of salivary gland-related contamination during haemolymph extraction was ruled out since tissue collection was carried out independently. Firstly, the haemolymph was collected; the insect was kept alive during such procedure and only a cut in one of its hind legs was needed, as mentioned in the methodology, so that the salivary glands suffered no risk of rupture during haemolymph collection at any time (added to line 564).

R2: The Discussion has been expanded and it has now been mentioned that humoral factors can be secreted directly to the haemocoel or intestinal lumen as immune response components to eliminate potential pathogens acquired during feeding (Garcia *et al*., 2010). A recent study about lipocalins (Santos *et al*., 2022) mentioned that these proteins are present in other types of tissue and could have multiple functions. The authors suggested that central nervous system expressed lipocalins could act as neurotransmitters, as OBPs when expressed in antennas and as lipid transporters in the haemolymph (line 571-576).

- Line 547. Did the authors inoculate the Dm28c strain in the hemolymph of R. colombiensis to see how long the parasites survive?

R: No, only in vitro experiments.

Reviewer #2: *In this manuscript, Barbosa et al. evaluated the lytic activity of haemolymph and saliva of R. prolixus and R. colombiensis against T. cruzi. The authors based their conclusions in experiments with the DM28c (TcI) and Y (TcII) strains, where the heamolymph of R. prolixus, but not R. colombiensis was capable of lysing both epimastigotes and tripomastigotes of the Y strain, while those of the Dm28c remained unaffected. Additionally, the authors report a lytic effect by R. prolixus saliva against Y strain epimastigotes. Finally, the authors performed a proteomic analyisis of haemolymph and saliva from the two vectors species to identify candidate lytic proteins. Unfortunately, I feel the data presented is not sufficient to support DTU level differences, only differences for two specific strains (Dm29c and Y) can be established. Overextending the conclusions to encompass the entire DTUs TcI and TcII is not appropriate.*

R: The title has been modified to read, “Comparative proteomic analysis of *Rhodnius prolixus* and *R. colombiensis* haemolymph and salivary glands has revealed candidates associated with differential lytic activity against *Trypanosoma cruzi* Dmc28c and *T. cruzi* Y”. TcI has been replaced by Dmc28c and TcII by Y throughout the text.

PLOS authors have the option to publish the peer review history of their article (what does this mean?). If published, this will include your full peer review and any attached files.

If you choose “no”, your identity will remain anonymous but your review may still be made public.

Do you want your identity to be public for this peer review? For information about this choice, including consent withdrawal, please see our Privacy Policy.

Reviewer #1: No

Reviewer #2: No
